# Multiple imputation for handling missing outcome data when estimating the relative risk

**DOI:** 10.1186/s12874-017-0414-5

**Published:** 2017-09-06

**Authors:** Thomas R. Sullivan, Katherine J. Lee, Philip Ryan, Amy B. Salter

**Affiliations:** 1 The University of Adelaide, School of Public Health, Adelaide, SA Australia; 2 Clinical Epidemiology and Biostatistics Unit, Murdoch Childrens Research Institute, Melville, VIC Australia; 3 Department of Paediatrics, University of Melbourne, Melbourne, VIC Australia

**Keywords:** Relative risk, Missing data, Multiple imputation

## Abstract

**Background:**

Multiple imputation is a popular approach to handling missing data in medical research, yet little is known about its applicability for estimating the relative risk. Standard methods for imputing incomplete binary outcomes involve logistic regression or an assumption of multivariate normality, whereas relative risks are typically estimated using log binomial models. It is unclear whether misspecification of the imputation model in this setting could lead to biased parameter estimates.

**Methods:**

Using simulated data, we evaluated the performance of multiple imputation for handling missing data prior to estimating adjusted relative risks from a correctly specified multivariable log binomial model. We considered an arbitrary pattern of missing data in both outcome and exposure variables, with missing data induced under missing at random mechanisms. Focusing on standard model-based methods of multiple imputation, missing data were imputed using multivariate normal imputation or fully conditional specification with a logistic imputation model for the outcome.

**Results:**

Multivariate normal imputation performed poorly in the simulation study, consistently producing estimates of the relative risk that were biased towards the null. Despite outperforming multivariate normal imputation, fully conditional specification also produced somewhat biased estimates, with greater bias observed for higher outcome prevalences and larger relative risks. Deleting imputed outcomes from analysis datasets did not improve the performance of fully conditional specification.

**Conclusions:**

Both multivariate normal imputation and fully conditional specification produced biased estimates of the relative risk, presumably since both use a misspecified imputation model. Based on simulation results, we recommend researchers use fully conditional specification rather than multivariate normal imputation and retain imputed outcomes in the analysis when estimating relative risks. However fully conditional specification is not without its shortcomings, and so further research is needed to identify optimal approaches for relative risk estimation within the multiple imputation framework.

**Electronic supplementary material:**

The online version of this article (10.1186/s12874-017-0414-5) contains supplementary material, which is available to authorized users.

## Background

The relative risk is a summary measure of effect for binary outcomes that is often of interest in medical research [1–4]. Unlike the odds ratio, the relative risk is simple to interpret and collapsible across covariate strata [5]. For rare outcomes, relative risks may be estimated from logistic regression models, since the odds ratio approximates the relative risk in this case [4]. For more common outcomes, the odds ratio overestimates the relative risk and so alternatives to logistic regression are required to estimate the relative risk. A standard approach to estimating the relative risk directly is to fit a generalized linear model with a binomial error distribution and a log link, known as the log binomial model [6, 7]. Since the log link allows predicted probabilities greater than one, convergence problems with this model are not uncommon, particularly for models containing continuous covariates or outcomes with high prevalence [6, 7]. Several alternative approaches to relative risk estimation have been proposed to address failed convergence with the log binomial model, with modified Poisson regression using a log link and a robust error variance [8] one of the more commonly used methods.

A common feature of epidemiologic investigations is the occurrence of missing data, which can result in biased and inefficient parameter estimates if inadequately handled during the statistical analysis. Among the more rigorous approaches to handling missing data, multiple imputation (MI) [9] has been widely adopted due to its flexibility and availability in statistical software packages [10]. MI involves fitting a statistical model to the observed data to estimate values for the missing data. To incorporate missing data uncertainty, multiple values are imputed for each missing observation, producing multiple complete datasets. Following analysis, parameter estimates from the multiple datasets are appropriately combined to give a single MI estimate. Standard implementations of MI assume that data are missing at random (MAR), which occurs when the probability of missing data depends only on observed data [11]. Provided this assumption is met and statistical models used for imputation and analysis are correctly specified, MI produces consistent and asymptotically efficient parameter estimates [9].

For arbitrary patterns of missing data (i.e. missing data occurring in any variable, in any pattern across variables), the two standard model-based methods of MI are fully conditional specification (FCS) [12–14], also known as chained equations, and multivariate normal imputation (MVNI) [15]. FCS involves specifying a series of univariate imputation models, one for each variable with missing data. Standard software uses logistic regression to impute incomplete binary outcomes, which assumes a linear relationship between the log odds of the risk and other variables in the imputation model. Incomplete covariates can similarly be imputed using appropriate univariate models (e.g. linear regression for continuous covariates). In contrast, MVNI assumes that all variables in the imputation model follow a multivariate normal distribution. For incomplete binary outcomes, an additional rounding step is also required following MVNI to convert continuous imputed values to binary values suitable for analysis [16]. Although FCS and MVNI have been evaluated in settings where the goal is to estimate the odds ratio using logistic regression [12, 16, 17], little is known about their performance when the aim is to estimate the relative risk. Importantly, it is unclear whether imputing outcomes using logistic regression in FCS or under a multivariate normal assumption in MVNI could lead to biased or inefficient estimation when the analysis involves a log binomial model.

A popular alternative to the standard implementation of MI for handling missing data in both outcome and exposure variables is the “multiple imputation, then deletion” approach (MID), where observations with imputed outcomes are excluded from the analysis [18]. Although MID is not advisable when the imputation model contains auxiliary variables for the outcome (i.e. variables that are not part of the analysis but which help to predict missing outcome values) [19], the approach can offer small efficiency gains over standard MI when imputation and analysis models are the same. Of relevance to the estimation of relative risks, it has been argued that removing imputed outcomes prior to analysis can help to minimize the bias introduced by a misspecified imputation model for the outcome [18]. Should the imputation of incomplete binary outcomes using FCS or MVNI lead to biased estimation of the relative risk, this claimed strength of MID could lessen this bias.

This article aims to (i) evaluate the performance of FCS and MVNI for handling missing outcome data when estimating the relative risk, and (ii) investigate whether deleting imputed outcomes prior to analysis improves the performance of FCS and MVNI in this setting. The rest of the article is set out as follows. In the next section, we describe the methods of FCS and MVNI in more detail, drawing attention to potential limitations. This is followed by an outline of the simulation methods used to address the article aims, and a summary of the simulation results. Finally, we conclude the article by discussing key findings and providing recommendations for practice.

## Methods

### Fully conditional specification

FCS involves specifying a series of univariate imputation models, one for each variable with missing data [12–14], with models tailored according to the distribution of the variable being imputed. For each variable with missing data, the FCS algorithm begins by replacing missing values with randomly selected observed values or the mean value for the same variable. Imputations are then generated by estimating each univariate model in turn, restricted to participants with observed values for the variable being considered and using imputed values for other variables; at each stage missing values are replaced by draws from their posterior predictive distribution. This process continues until all incomplete variables have been imputed and is repeated several times in order to stabilise results, leading to the generation of a single imputed dataset. Additional imputed datasets are obtained by independently repeating this process.

Despite its flexibility, FCS is not without limitations. One concern with the approach is the possibility of specifying univariate imputation models where the conditional distributions implied do not correspond to a valid joint distribution. A potential consequence of this is that results could vary according to the ordering of univariate imputation models within the FCS procedure. Fortunately this issue appears to have little impact on results in practice [12, 13, 17, 20]. Another drawback of FCS is that it can be time consuming to implement in settings containing a large number of incomplete variables, since univariate imputation models need to be specified for each incomplete variable in the imputation model.

### Multivariate normal imputation

MVNI is a joint modelling approach to imputation where all variables in the imputation model are assumed to follow a multivariate normal distribution. First implemented by Schafer [15], MVNI uses a Markov chain Monte Carlo algorithm (known as data augmentation) for imputation. Initially, missing values are imputed based on assumed starting parameter values for the multivariate normal distribution. These are typically obtained from available data using the expectation-maximisation algorithm. Next, updated parameter values for the multivariate normal distribution are drawn from their posterior distribution based on the observed and imputed data. This iterative process of imputing missing values and drawing updated parameter values continues until these values converge to a stationary distribution [15, 21]. Following these “burn-in” iterations, a set of imputed values is taken. In order to reduce dependence between imputations, additional iterations are performed before the next set of imputed values is obtained.

Due to its strong theoretical underpinnings, MVNI is an appealing method when multivariate normality holds, but such an assumption is not always realistic, particularly when the imputation model contains binary variables. Although several authors have reported good performance with MVNI for binary variables [15, 16, 20, 22], it remains difficult to make global statements about the robustness of this approach to violations of multivariate normality, whether in the specific case of binary variables or more generally.

### Simulation study

The performance of FCS and MVNI for handling missing outcome data when estimating the relative risk was evaluated using data simulation. In order to attribute any deficiencies in performance to the method of MI, rather than getting caught up in complexities of the data, we focused on relatively simple simulation scenarios. In each simulation scenario, 2000 datasets of size *n* = 1000 were generated from the log binomial model log*P*(*Y* = 1) = *β*_0_ + *β*_1_ *X*_1_ + *β*_2_ *X*_2_, where *X*_1_ and *X*_2_ were binary or normally distributed exposure variables and *Y* was the binary outcome. A relatively large sample size was chosen to avoid zero cells in cross-tabulations involving the outcome. Following generation of complete datasets, values in *X*_2_ and *Y* were set to missing according to a specified MAR mechanism to produce an arbitrary pattern of missing data in these two variables. Missing values were then multiply imputed using FCS or MVNI with *m* = 20 imputations. For FCS, missing values in *Y* were imputed using a logistic regression model, while imputations for binary or normally distributed *X*_2_ were generated from a logistic or linear regression model respectively. A total of 20 cycles were used for each imputation, with the outcome imputed last. For MVNI, missing values were imputed using a Markov chain Monte Carlo algorithm with a burn-in of 200 iterations. Following imputation with MVNI, imputed values in the outcome were rounded to binary values using adaptive rounding, which has been recommended over alternative rounding techniques [16]. Finally, complete datasets either retaining or deleting imputed outcomes were analyzed using log binomial models (or modified Poisson regression as appropriate), with parameter estimates for *β*_1_ and *β*_2_ combined across datasets using Rubin’s rules [9]. Since the outcome *Y* was generated under the analysis model, any deficiencies in performance could be attributed to the method of MI. For reference, a complete case analysis (CCA) restricted to participants with complete data on both *Y* and *X*_2_ was also performed in each simulation scenario.

### Simulation study 1: Categorical exposures

In simulation study 1, *X*_1_ and *X*_2_ were generated as binary variables with a prevalence of 0.50 and a relative risk for their association (RR(*X*_1_, *X*_2_)) of 2 or 3, to induce moderate or strong confounding respectively. In simulating values for the outcome *Y*, *β*_1_ and *β*_2_ were both set to log(2) or log(3) to give conditional relative risks (i.e. RR(*Y*, *X*_1_| *X*_2_) and RR(*Y*, *X*_2_| *X*_1_)) of 2 or 3. Lastly the intercept *β*_0_ was chosen to give an overall outcome prevalence of 0.10 or 0.30. Following generation of complete datasets, values in *Y* and *X*_2_ were set to missing according to one of two MAR mechanisms:

1. Coordinated: logit *P*(*Y* missing) = logit *P*(*X*_2_ missing) = *α* + *λX*_1_.
2. Opposite: logit *P*(*Y* missing) = *α* + *λX*_1_ ,  logit *P*(*X*_2_ missing) = *α* + *λ*(1 − *X*_1_).

Under the coordinated mechanism, participants with missing data were often missing both *Y* and *X*_2_, whereas under the opposite mechanism, participants with missing data tended to be missing either *Y* or *X*_2_ (but not both). For both mechanisms, the parameter *λ* was set to 1 or 2 to indicate a moderate or strong missing data mechanism respectively, while *α* was chosen to produce 30% missing data in *Y* and *X*_2_. Collectively this resulted in 4 missing data patterns and 32 simulation scenarios. Following imputation, complete datasets were analyzed using log binomial models. Provided MVNI was applied with adaptive rounding for imputed values in *X*_2_ (in addition to *Y*), there were no convergence issues with the log binomial model in this setting.

### Simulation study 2: Continuous exposures

For simulation study 2, *X*_1_ and *X*_2_ were generated from a bivariate normal distribution with mean 0, variance 0.20 and correlation (corr(*X*_1_, *X*_2_)) 0.30 or 0.70. Again *β*_1_ and *β*_2_ were set to log(2) or log(3) to give conditional relative risks of 2 or 3, while *β*_0_ was chosen to give an outcome prevalence of 0.10 or 0.30. One concern when simulating data under a log binomial model with unbounded continuous covariates is the possibility of generating ‘success’ probabilities greater than one. In choosing the variance for *X*_1_ and *X*_2_, we sought to maximize the size of standardized conditional relative risks while minimizing the occurrence of invalid success probabilities. With a variance of 0.20, invalid success probabilities were rare, except in settings involving an outcome prevalence of 0.30 and conditional relative risks of 3 (where 5.4% of success probabilities exceeded one). Following previous simulation studies exploring the relative risk (e.g. [23]), *X*_1_ and *X*_2_ were resampled in these instances to ensure valid success probabilities.

Letting ${Z}_1={X}_1/\sqrt{\operatorname{var}\left({X}_1\right)}$, the coordinated and opposite missing data mechanisms were adapted for the continuous setting as follows:

1. Coordinated: logit *P*(*Y* missing) = logit *P*(*X*_2_ missing) = *α* + *λZ*_1_.
2. Opposite: logit *P*(*Y* missing) = *α* + *λZ*_1_ ,  logit *P*(*X*_2_ missing) = *α* − *λZ*_1_.

In line with simulation study 1, *λ* was set to 1 or 2 and *α* was chosen to produce 30% missing data in *Y* and *X*_2_. Again this resulted in 4 missing data patterns and 32 simulation scenarios. As non-convergence with the log binomial model was a considerable problem in this setting, often occurring for some but not all imputed datasets within a single simulation, we elected to analyze all complete datasets using modified Poisson regression.

### Comparisons

The performance of the MI approaches in estimating parameters *β*_1_ and *β*_2_ was evaluated in terms of bias (average difference between estimate and true value) and the coverage of estimated 95% confidence intervals (proportion of 95% confidence intervals containing the true value). With 2000 simulated datasets per simulation scenario, on 95% of occasions the coverage is expected to lie between 0.94 and 0.96 for a true coverage of 0.95. For each parameter, the average within-simulation estimated standard error (denoted the average standard error), the standard error of parameter estimates across simulated datasets (denoted the empirical standard error), and the mean square error (average squared difference between the estimate and the true value) were also derived. All analyses were performed in SAS version 9.4 (SAS Institute, Inc., Cary, North Carolina). Multiple imputation was carried out using the MI procedure, while analysis was performed using the GENMOD and MIANALYZE procedures. The SAS code for implementing the simulation study is available in the Additional file 1: Web Appendix.

## Results

### Simulation study 1: Categorical exposures

Table 1 displays results for the categorical exposure setting in scenarios with a strong missing data mechanism (*λ* = 2), where RR(*X*_1_, *X*_2_) = 2 and *β*_1_ = *β*_2_ = log(3). Similar results were observed for RR(*X*_1_, *X*_2_) = 3, while absolute biases of the imputation approaches were smaller in magnitude when *λ* = 1 and *β*_1_ = *β*_2_ = log(2). Full results for all simulation scenarios are available in the Additional file 1: Web Appendix. MVNI performed poorly across the 32 simulation scenarios, consistently producing estimates of *β*_2_ that were biased towards the null (bias range − 0.32 to −0.10). The bias of −0.32 shown in Table 1 for an outcome prevalence of 0.30 under the coordinated mechanism equates to a relative risk estimate of 2.19 compared with the true value of 3; coverage was just 0.55 in this scenario. Bias was less of a concern for *β*_1_ (bias range − 0.08 to 0.07). Deleting imputed outcomes following MVNI led to some reduction in absolute bias for *β*_2_, although estimates for *β*_1_ were moderately biased away from the null with this approach (bias range 0.02 to 0.11). Interestingly, average and empirical standard errors were noticeably increased by the deletion of imputed outcomes following MVNI. Compared to MVNI (without deletion), MVNI with deletion led to small increases in the mean square error for *β*_1_, but tended to decrease the mean square error for *β*_2_.

**Table 1:** Results for *X*_1_ and *X*_2_ Binary, *λ* = 2, RR(*X*_1_, *X*_2_) = 2 and *β*_1_ = *β*_2_ = log(3)

Simulation scenario	Method	*β*_1_					*β*_2_				
		Bias	Avg SE	Emp SE	Coverage	MSE	Bias	Avg SE	Emp SE	Coverage	MSE
Coordinated, prevalence =0.10	MVNI	−0.08	0.30	0.28	0.951	0.08	−0.28	0.35	0.28	0.896	0.15
	MVNI + deletion	0.06	0.30	0.31	0.955	0.10	−0.09	0.39	0.35	0.956	0.13
	FCS	0.02	0.30	0.31	0.955	0.10	0.00	0.40	0.40	0.962	0.16
	FCS + deletion	0.02	0.30	0.31	0.948	0.09	0.01	0.40	0.40	0.962	0.16
	CCA	0.01	0.34	0.34	0.953	0.12	0.03	0.40	0.40	0.964	0.16
Coordinated, prevalence =0.30	MVNI	0.03	0.16	0.15	0.952	0.02	−0.32	0.17	0.16	0.547	0.13
	MVNI + deletion	0.05	0.16	0.16	0.948	0.03	−0.15	0.20	0.19	0.872	0.06
	FCS	0.03	0.16	0.16	0.951	0.03	−0.11	0.20	0.21	0.893	0.05
	FCS + deletion	0.02	0.16	0.16	0.955	0.02	−0.06	0.21	0.21	0.932	0.05
	CCA	0.01	0.17	0.17	0.953	0.03	0.01	0.21	0.22	0.949	0.05
Opposite, prevalence =0.10	MVNI	−0.08	0.29	0.28	0.949	0.08	−0.26	0.34	0.26	0.908	0.13
	MVNI + deletion	0.05	0.30	0.30	0.955	0.09	−0.07	0.37	0.33	0.964	0.11
	FCS	0.01	0.30	0.31	0.952	0.10	0.03	0.39	0.39	0.963	0.16
	FCS + deletion	0.01	0.30	0.31	0.950	0.09	0.05	0.39	0.40	0.964	0.16
	CCA	0.03	0.39	0.41	0.956	0.17	0.03	0.39	0.39	0.965	0.15
Opposite, prevalence =0.30	MVNI	0.00	0.00	0.15	0.961	0.02	−0.20	0.18	0.16	0.805	0.06
	MVNI + deletion	0.03	0.16	0.15	0.961	0.02	−0.02	0.20	0.19	0.952	0.03
	FCS	0.00	0.16	0.16	0.951	0.02	0.01	0.20	0.20	0.948	0.04
	FCS + deletion	−0.02	0.16	0.15	0.949	0.02	0.07	0.21	0.21	0.947	0.05
	CCA	0.01	0.20	0.20	0.952	0.04	0.02	0.20	0.20	0.952	0.04

*Abbreviations: MVNI* multivariate normal imputation, *FCS* fully conditional specification, *CCA* complete case analysis, *Avg SE* average standard error, *Emp SE* empirical standard error, *MSE* mean square error

In contrast to MVNI, FCS performed fairly well for categorical exposures, with absolute bias only exceeding 0.10 for the coefficient *β*_2_ in scenarios involving a strong coordinated mechanism, an outcome prevalence of 0.30 and where *β*_1_ = *β*_2_ = log(3). Excluding simulation scenarios where the bias for *β*_2_ exceeded 0.10, the coverage of estimated 95% confidence intervals for *β*_1_ and *β*_2_ remained close to nominal levels (range 0.93 to 0.96). Compared to FCS (without deletion), FCS with deletion led to small reductions in absolute bias for *β*_2_ under the coordinated mechanism for an outcome prevalence of 0.30, but slight increases in absolute bias under the opposite mechanism for the same outcome prevalence. There was little difference in average standard errors, empirical standard errors, and mean square errors between FCS and FCS with deletion, although both approaches were less precise than MVNI.

Interestingly, CCA exhibited little bias in simulation scenarios involving categorical exposures, with a maximum absolute bias of 0.06 for both *β*_1_ and *β*_2_. As expected, in discarding information from partially observed cases, CCA was noticeably less efficient than the MI approaches, especially for the coefficient *β*_1_ for the fully observed exposure *X*_1_.

### Simulation study 2: Continuous exposures

To ensure that any deficiencies in performance in the continuous exposure setting could be attributed to the method of MI and not the use of modified Poisson regression for estimating relative risks, the accuracy of this method was first verified in complete datasets (i.e. before values in *Y* and *X*_2_ were set to missing). Reassuringly, unbiased estimates for *β*_1_ and *β*_2_ were observed across all simulation scenarios (absolute bias ≤0.01), with estimated 95% confidence intervals demonstrating appropriate coverage (i.e. within the range 0.94 to 0.96) (results not shown).

The performance deficits of MI were more pronounced in the presence of continuous exposures than categorical exposures. Table 2 shows results for scenarios with a strong missing data mechanism (*λ* = 2), where corr(*X*_1_, *X*_2_) = 0.70 and *β*_1_ = *β*_2_ = log(3). A similar pattern of results was observed in other simulation scenarios, although absolute biases were smaller in magnitude for *λ* = 1 and *β*_1_ = *β*_2_ = log(2). As shown in Table 2, MVNI produced estimates for *β*_1_ and *β*_2_ that were biased towards the null, with the largest absolute bias observed for *β*_1_ under the opposite mechanism with an outcome prevalence of 0.10 (relative risk estimate of 1.68 compared with the true value of 3). Across all 32 simulation scenarios, the median bias of MVNI was −0.21 for *β*_1_ (range − 0.58 to −0.10) and −0.12 for *β*_2_ (range − 0.27 to −0.06). Deleting imputed outcomes following MVNI reduced the bias of this imputation method, although moderate bias remained for *β*_2_ in scenarios with an outcome prevalence of 0.30. The cost of this bias reduction was substantially larger average standard errors in comparison to MVNI. In terms of accuracy, deleting imputed outcomes following MVNI led to reductions in the mean square error relative to MVNI without deletion in 26/32 and 12/32 simulation scenarios for *β*_1_ and *β*_2_ respectively.

**Table 2:** Results for *X*_1_ and *X*_2_ Continuous, *λ* = 2, Corr(*X*_1_, *X*_2_) = 0.70 and *β*_1_ = *β*_2_ = log(3)

Simulation scenario	Method	*β*_1_					*β*_2_				
		Bias	Avg SE	Emp SE	Coverage	MSE	Bias	Avg SE	Emp SE	Coverage	MSE
Coordinated, prevalence =0.10	MVNI	−0.56	0.48	0.39	0.838	0.47	−0.22	0.50	0.43	0.958	0.24
	MVNI + deletion	0.01	0.58	0.55	0.965	0.30	−0.03	0.55	0.53	0.959	0.28
	FCS	−0.08	0.51	0.49	0.961	0.25	−0.14	0.50	0.48	0.950	0.25
	FCS + deletion	0.02	0.58	0.55	0.964	0.30	−0.04	0.55	0.53	0.961	0.28
	CCA	0.01	0.66	0.67	0.943	0.45	0.01	0.53	0.55	0.936	0.30
Coordinated, prevalence =0.30	MVNI	−0.26	0.26	0.20	0.890	0.11	−0.27	0.25	0.20	0.859	0.11
	MVNI + deletion	0.01	0.31	0.26	0.978	0.07	−0.11	0.28	0.23	0.963	0.07
	FCS	−0.09	0.26	0.22	0.962	0.06	−0.24	0.24	0.21	0.878	0.10
	FCS + deletion	0.02	0.31	0.26	0.980	0.07	−0.12	0.28	0.23	0.963	0.07
	CCA	0.02	0.32	0.32	0.951	0.11	0.00	0.25	0.26	0.950	0.07
Opposite, prevalence =0.10	MVNI	−0.58	0.47	0.37	0.830	0.47	−0.17	0.48	0.42	0.961	0.21
	MVNI + deletion	0.00	0.56	0.52	0.966	0.28	0.02	0.53	0.51	0.959	0.26
	FCS	−0.08	0.48	0.46	0.961	0.22	−0.07	0.49	0.47	0.959	0.22
	FCS + deletion	0.01	0.56	0.52	0.971	0.27	0.01	0.53	0.51	0.962	0.26
	CCA	0.00	0.60	0.62	0.939	0.39	0.01	0.48	0.50	0.938	0.25
Opposite, prevalence =0.30	MVNI	−0.25	0.24	0.19	0.886	0.10	−0.07	0.26	0.20	0.981	0.05
	MVNI + deletion	0.01	0.30	0.25	0.983	0.06	0.06	0.29	0.23	0.980	0.06
	FCS	−0.07	0.24	0.21	0.974	0.05	−0.02	0.26	0.22	0.980	0.05
	FCS + deletion	0.02	0.29	0.25	0.983	0.06	0.05	0.29	0.23	0.982	0.06
	CCA	0.00	0.29	0.29	0.945	0.08	0.01	0.23	0.22	0.949	0.05

*Abbreviations: MVNI* multivariate normal imputation, *FCS* fully conditional specification, *CCA* complete case analysis, *Avg SE* average standard error, *Emp SE* empirical standard error, *MSE* mean square error

FCS also produced estimates of *β*_1_ and *β*_2_ that were biased towards the null, albeit to a lesser degree than MVNI. The bias of −0.24 shown in Table 2 for an outcome prevalence of 0.30 under the coordinated mechanism translates to a relative risk estimate of just 2.37 versus the true value of 3. In addition to the more extreme simulation scenarios, noticeable bias for *β*_2_ (absolute bias >0.10) was apparent in simulation scenarios with an outcome prevalence of 0.10, a moderate missing data mechanism or where *β*_1_ = *β*_2_ = log(2). Deleting imputed outcomes following FCS tended to decrease the bias of this imputation approach, with absolute bias reduced in 28/32 and 26/32 simulation scenarios for *β*_1_ and *β*_2_ respectively. The trade-off for this bias reduction was a substantial loss in precision. Across the 32 simulation scenarios, average standard errors were 14.4% larger for *β*_1_ and 8.1% larger for *β*_2_ with the deletion of imputed outcomes following FCS compared to FCS alone. A consequence of the substantial loss in precision with the deletion of imputed outcomes following FCS was a loss in overall accuracy, with the mean square error increased relative to FCS without deletion in 30/32 and 26/32 simulation scenarios for *β*_1_ and *β*_2_ respectively.

Another noteworthy result from the continuous exposure setting was that average standard errors were consistently larger than empirical standard errors. Averaged across the 32 simulation scenarios, average standard errors for *β*_1_ and *β*_2_ were 25.8% and 17.9% larger than empirical standard errors respectively for MVNI, 14.4% and 11.9% larger for MVNI with deletion, 10.4% and 9.5% larger for FCS, and 14.3% and 12.1% larger for FCS with deletion. Discrepancies were most prominent in simulation scenarios with an outcome prevalence of 0.30. In scenarios where *β*_1_ and *β*_2_ were estimated with little bias, coverage probabilities also tended to be much higher than the nominal level of 0.95. Collectively these results suggest that estimated confidence intervals were too wide.

As observed for categorical exposures, CCA exhibited little bias but tended to produce inefficient estimates of *β*_1_ in scenarios involving continuous exposures. Interestingly, CCA produced more precise estimates of *β*_2_ than the two MID approaches; across the 32 simulation scenarios, average standard errors for *β*_2_ were 9.3% smaller with CCA relative to both deletion approaches.

### Sensitivity analyses

In light of the relatively poor performance of the MI approaches for relative risk estimation, we undertook additional analyses to explore whether findings were sensitive to choices made during the fitting of imputation models or to the simulation parameters considered. First, we investigated the performance of simple rounding following MVNI as an alternative to adaptive rounding. While differences were minimal in most scenarios, MVNI introduced slightly more bias in both categorical and continuous exposure settings when simple rounding was used in place of adaptive rounding (results not shown). Next, we investigated the performance of FCS with the outcome imputed before rather than after the incomplete covariate *X*_2_. This modification made little difference to results (also not shown). We then explored the performance of the four MI approaches in scenarios involving *n* = 250 rather than *n* = 1000 observations. Excluding simulation scenarios with binary *X*_1_ and *X*_2_ where the reduced sample size resulted in zero cells in cross-tabulations involving the outcome (i.e. where log binomial analysis models would not converge), this change made little difference to the bias and coverage of parameter estimates (results not shown).

To investigate whether biased estimation would persist if the exposures were independent of one another, if the outcome was unrelated to one or both exposures, or if data were missing completely at random (i.e. probability of missing data unrelated to observed or unobserved data), several “null-case” simulation settings were considered. Table 3 shows results for continuous *X*_1_ and *X*_2_ under the coordinated missing data mechanism for an outcome prevalence of 0.30. The reference case for comparisons in this table was the previously considered simulation scenario involving a strong missing data mechanism (*λ* = 2), where corr(*X*_1_, *X*_2_) = 0.70 and *β*_1_ = *β*_2_ = log(3). As shown in the table, the four MI approaches continued to produce biased parameter estimates when the exposures were independent of one another (i.e. corr(*X*_1_, *X*_2_) = 0). When the outcome was unrelated to one of the exposures, parameter estimates remained biased only for the exposure that was predictive of the outcome; little bias was observed with any of the MI approaches when both exposures were unrelated to the outcome. Lastly, bias was reduced but still evident when data were missing completely at random. A similar pattern of results was observed with binary *X*_1_ and *X*_2_, and for an outcome prevalence of 0.10. Full results for these sensitivity analyses are available in the Additional file 1: Web Appendix.

**Table 3:** Bias in Scenarios with *X*_1_ and *X*_2_ Continuous, Coordinated Missing Data Mechanism and Outcome Prevalence = 0.30

Simulation scenario	Parameter	MVNI	MVNI + deletion	FCS	FCS + deletion
1. Corr(*X*_1_, *X*_2_) = 0.70, *β*_1_ = *β*_2_ = log(3), *λ* = 2	*β*_1_	−0.26	0.01	−0.09	0.02
	*β*_2_	−0.27	−0.11	−0.24	−0.12
2. As in (1.), but with Corr(*X*_1_, *X*_2_) = 0	*β*_1_	−0.27	−0.05	−0.15	−0.05
	*β*_2_	−0.21	−0.06	−0.16	−0.06
3. As in (1.), but with *β*_1_ = 0	*β*_1_	−0.04	0.02	0.01	0.02
	*β*_2_	−0.17	−0.03	−0.10	−0.03
4. As in (1.), but with *β*_2_ = 0	*β*_1_	−0.24	0.00	−0.10	0.00
	*β*_2_	0.00	0.00	0.00	0.00
5. As in (1.), but with *β*_1_ = *β*_2_ = 0	*β*_1_	−0.01	−0.01	−0.01	−0.01
	*β*_2_	0.01	0.01	0.01	0.01
6. As in (1.), but with *λ* = 0 (MCAR)	*β*_1_	−0.11	0.00	0.00	0.00
	*β*_2_	−0.17	−0.08	−0.08	−0.08

*Abbreviations: Corr* correlation, *MCAR* missing completely at random, *MVNI* multivariate normal imputation, *FCS* fully conditional specification

Lastly, to evaluate whether the performance deficiencies of FCS could be attributed solely to the misspecified logistic imputation model for the outcome, we considered additional simulation scenarios where missing data were restricted to either *Y* or *X*_2_ only (with logit *P*(*missing*) = *α* + *λX*_1_). Since data were missing in a single variable, missing values were imputed 20 times using logistic or linear regression as appropriate. Table 4 shows results for an outcome prevalence of 0.30, *λ* = 2 and *β*_1_ = *β*_2_ = log(3) for categorical exposures with RR(*X*_1_, *X*_2_) = 2 or continuous exposures with corr(*X*_1_, *X*_2_) = 0.70. The results for the original simulation scenario for FCS under the coordinated mechanism are also presented for comparison. As shown in the table, estimation remained biased when missing data were restricted to *X*_2_. Indeed for continuous exposures, the bias for *β*_2_ was larger when missing data were restricted to *X*_2_ compared to when missing data were restricted to *Y*. Thus it seems that the shortcomings of FCS were at least partly attributable to the choice of conditional imputation model for the incomplete covariate *X*_2_. It is worth noting that the bias following the imputation of continuous *X*_2_ with a univariate linear model, as shown in Table 4, also suggests that the performance deficits seen with MVNI in the continuous exposure setting were partly due to inappropriate imputed values in the exposure (and not just the outcome).

**Table 4:** Bias in Scenarios with *λ* = 2, Outcome Prevalence = 0.30 and *β*_1_ = *β*_2_ = log(3)

Simulation scenario	Parameter	Coordinated missing data in *Y* and *X*_2_ (FCS)	Missing data in *Y* only	Missing data in *X*_2_ only
Binary *X*_1_ and *X*_2_, RR(*X*_1_, *X*_2_) = 2	*β*_1_	0.03	0.02	0.02
	*β*_2_	−0.11	−0.06	−0.05
Continuous *X*_1_ and *X*_2_, Corr(*X*_1_, *X*_2_) = 0.70	*β*_1_	−0.09	−0.08	−0.03
	*β*_2_	−0.24	−0.07	−0.22

*Abbreviations: RR* relative risk, *Corr* correlation, *FCS* fully conditional specification

## Discussion

Given the widespread use of MI and the popularity of the relative risk, the lack of research on the application of MI for estimating the relative risk is surprising. In this study we demonstrated that standard model-based methods of MI can produce biased estimates of the relative risk with overly wide confidence intervals when data are MAR. Performance deficits were particularly evident when the analysis included continuous exposures, and in settings with larger relative risks, stronger missing data mechanisms and higher outcome prevalences. These findings raise concerns about the use of standard MI methods for relative risk estimation.

The primary aim of this study was to contrast the performance of MVNI and FCS for handling missing outcome data when estimating the relative risk. MVNI performed more poorly than FCS, producing relative risk estimates that were often substantially biased towards the null, both for categorical and continuous exposures. Although MVNI has been shown to be robust to violations of the multivariate normal assumption across a range of other settings, for example in estimating odds ratios or dealing with non-normal exposure variables [16, 20], such robustness to imputation model misspecification was not evident here. In contrast, FCS performed well when the analysis involved categorical exposures, only introducing noticeable bias for an outcome prevalence of 0.30, a strong missing data mechanism and large relative risks. Performance was less satisfactory in the presence of continuous exposures, with noticeable bias towards the null also evident in settings involving moderate relative risks or an outcome prevalence of 0.10. Even when relative risks for continuous exposures were estimated with little bias, FCS produced confidence intervals that were too wide. While we would recommend FCS over MVNI for relative risk estimation based on the simulation results presented here, clearly the approach is not without its shortcomings.

The secondary aim of this study was to evaluate whether deleting imputed outcomes improves the performance of MI for relative risk estimation. Focusing on FCS as the better performed method of MI, we observed little difference between FCS with and without deletion of imputed outcomes for analysis models involving categorical exposures. In the presence of continuous exposures, deleting imputed outcomes following FCS was associated with partial decreases in absolute bias at the expense of large increases in average standard errors; an interesting finding given that deletion improves the precision of estimation in settings where analysis and imputation models are the same [18]. The lost precision with MID in the continuous exposure settings suggests that imputed values in the outcome contained information that was useful for analysis, which may be due to inconsistencies between the imputation and analysis models. Of course, since the imputation model was misspecified, this additional information (from the imputed outcome values) could also result in increased bias in a conventional MI analysis. In any case, we find it difficult to recommend MID for relative risk estimation based on these results, particularly since the approach is only advisable in settings where auxiliary variables for the outcome are unavailable [19].

Although logistic regression is the standard choice for imputing binary outcomes in software for implementing FCS, evidently this model is not optimal for relative risk estimation. Since controlling for confounding differs between the odds ratio and the relative risk [24], it is perhaps unsurprising that performance deficits were observed with FCS in this simulation study. This raises the question of whether an alternative conditional imputation model for the outcome should be adopted with FCS when relative risk estimation of interest. Assuming the analysis model is appropriately specified, an obvious candidate to minimise the problems of imputation model misspecification is the log binomial model, however issues with non-convergence could be a significant limitation in the context of FCS. As relative risks are often estimated using modified Poisson regression, another possibility would be to impute outcomes using Poisson regression. One difficulty with this approach is that imputed outcome values would be counts and would thus entail the use of modified Poisson regression in the analysis or the use of a rounding method prior to analysis with a log binomial model. Rounding methods have not been developed for this purpose. Another important challenge would be to incorporate a robust estimate of the error variance within the imputation model, since ordinary Poisson regression tends to overestimate the standard error for the relative risk [8]. Although other approaches have been proposed to estimate relative risks (e.g. Cox regression with constant time at risk [25]), like Poisson regression, they typically require the use of a robust error variance which would need to be accounted for during imputation. This is difficult to achieve with current MI software.

In addition to the misspecified logistic model for imputing the outcome, sensitivity analyses revealed that the bias introduced by FCS could also be attributed to the conditional models used to impute the covariates. Imputing the continuous covariate using linear regression in FCS assumed a linear relationship between the covariate and the outcome, which was inconsistent with the data generation model. A similar argument applies for the imputation of binary covariates using logistic regression. In a recent article, Bartlett and colleagues [26] proposed a modification to the standard FCS algorithm such that incomplete covariates are imputed from models that are compatible with the intended analysis model. While the approach seems promising in this context, further research is needed to understand its properties and suitability for relative risk estimation.

Due to convergence problems with the log binomial model in the continuous exposure setting, we elected to analyze all imputed datasets using the popular modified Poisson regression approach. Simulation results demonstrated that this method performed well in the absence of missing data, which is consistent with previous investigations of the method [8, 23, 25]. An interesting consideration that arose following imputation was whether to use modified Poisson regression to analyze all imputed datasets or only those datasets where the log binomial model failed to converge. We chose the former approach, as this was simpler to implement and seemed more in keeping with Rubin’s rules, however the latter could also be considered in future work.

Given the missing data mechanisms considered in the simulation study, it is not surprising that CCA produced parameter estimates with little bias. For missing data in a univariate outcome, CCA is known to produce unbiased and fully efficient estimates of regression coefficients when the probability of missing data depends only on fully observed covariates [27–29]. For missing data restricted to a covariate, CCA is known to be unbiased (but not fully efficient) if the probability of missing data is independent of the outcome conditional on the other covariates in the model [30]. Both of these conditions were satisfied in the simulation study, where the probability of missing data in *Y* and *X*_2_ depended only on the fully observed covariate *X*_1_. Clearly these conditions do not always hold in more complex practical settings, and CCA can introduce considerable bias when data are MAR. Taking into account the potential bias and inefficiency of CCA, we do not advocate its use over MI for handling arbitrary patterns of missing data when estimating the relative risk.

Although we anticipate similar deficits with MVNI and FCS in more complex practical settings, it is difficult to draw definitive conclusions from a restricted set of simulation scenarios. Further exploration of the performance of these MI methods in real datasets (where the missing data mechanism is unknown) and in simulation scenarios with different covariate characteristics, outcome prevalences and missing data mechanisms would certainly be useful. A further limitation of the current study is that we did not evaluate alternatives to standard model-based methods of MI for handling missing data. Most notably we did not consider inverse probability weighting, a method that involves weighting complete cases in the analysis according to the inverse of the probability of being a complete case [31]. We chose to focus on MI as it known to be more efficient than inverse probability weighting, particularly in the presence of auxiliary variables and for arbitrary patterns of missing data. However in light of the performance deficits of MI, further research could explore the use of inverse probability weighting in this setting. Within the MI framework, we did not consider less widely used model-based methods such as the general location model for mixtures of continuous and categorical variables, or non-parametric methods such as hot deck imputation. Again further research might consider the use of these approaches for relative risk estimation.

## Conclusions

In summary, standard model-based methods of MI can produce biased and inefficient estimates of the relative risk due to misspecification of the imputation model. Should MI be chosen to handle missing data, we recommend researchers avoid MVNI and instead use FCS without deletion for estimating relative risks. However, further research is needed to identify optimal approaches for relative risk estimation within the MI framework.

## Additional file

Additional file 1: Web Appendix. (DOCX 227 kb)

## Abbreviations

FCS: fully conditional specificationMAR: missing at random
MI: multiple imputation
MID: multiple imputation then deletion
MVNI: multivariate normal imputation

## References

[1] Greenland S (1987). Interpretation and choice of effect measures in epidemiologic analyses. Am J Epidemiol.

[2] Lee J (1994). Odds ratio or relative risk for cross-sectional data?. Int J Epidemiol.

[3] Sinclair JC, Bracken MB (1994). Clinically useful measures of effect in binary analyses of randomized trials. J Clin Epidemiol.

[4] McNutt LA, Wu C, Xue X, Hafner JP (2003). Estimating the relative risk in cohort studies and clinical trials of common outcomes. Am J Epidemiol.

[5] Cummings P (2009). The relative merits of risk ratios and odds ratios. Archives of Pediatrics & Adolescent Medicine.

[6] Wacholder S (1986). Binomial regression in GLIM: estimating risk ratios and risk differences. Am J Epidemiol.

[7] Skov T, Deddens J, Petersen MR, Endahl L (1998). Prevalence proportion ratios: estimation and hypothesis testing. Int J Epidemiol.

[8] Zou G (2004). A modified poisson regression approach to prospective studies with binary data. Am J Epidemiol.

[9] Rubin D (1987). Multiple imputation for nonresponse in surveys.

[10] Hayati Rezvan P, Lee KJ, Simpson JA (2015). The rise of multiple imputation: a review of the reporting and implementation of the method in medical research. BMC Med Res Methodol.

[11] Rubin D (1976). Inference and missing data. Biometrika.

[12] Raghunathan T, Lepkowski J, Van Hoewyk J, Solenberger P (2001). A multivariate technique for multiply imputing missing values using a sequence of regression models. Survey Methodology.

[13] van Buuren S (2007). Multiple imputation of discrete and continuous data by fully conditional specification. Stat Methods Med Res.

[14] White IR, Royston P, Wood AM (2011). Multiple imputation using chained equations: issues and guidance for practice. Stat Med.

[15] Schafer JL (1997). Analysis of incomplete multivariate data.

[16] Bernaards CA, Belin TR, Schafer JL (2007). Robustness of a multivariate normal approximation for imputation of incomplete binary data. Stat Med.

[17] van Buuren S, Brand J, Groothuis-Oudshoorn C, Rubin D (2006). Fully conditional specification in multivariate imputation. J Stat Comput Simul.

[18] von Hippel PT (2007). Regression with missing Ys: an improved strategy for analyzing multiply imputed data. Sociol Methodol.

[19] Sullivan TR, Salter AB, Ryan P, Lee KJ (2015). Bias and precision of the "multiple imputation, then deletion" method for dealing with missing outcome data. Am J Epidemiol.

[20] Lee KJ, Carlin JB (2010). Multiple imputation for missing data: fully conditional specification versus multivariate normal imputation. Am J Epidemiol.

[21] Schafer JL, Olsen MK (1998). Multiple imputation for multivariate missing-data problems: a data analyst's perspective. Multivar Behav Res.

[22] Romaniuk H, Patton GC, Carlin JB (2014). Multiple imputation in a longitudinal cohort study: a case study of sensitivity to imputation methods. Am J Epidemiol.

[23] Yelland LN, Salter AB, Ryan P (2011). Performance of the modified Poisson regression approach for estimating relative risks from clustered prospective data. Am J Epidemiol.

[24] Miettinen OS, Cook EF (1981). Confounding: essence and detection. Am J Epidemiol.

[25] Barros AJ, Hirakata VN (2003). Alternatives for logistic regression in cross-sectional studies: an empirical comparison of models that directly estimate the prevalence ratio. BMC Med Res Methodol.

[26] Bartlett JW, Seaman SR, White IR, Carpenter JR (2015). Multiple imputation of covariates by fully conditional specification: accommodating the substantive model. Stat Methods Med Res.

[27] Little RJA (1992). Regression with missing X's: a review. J Am Stat Assoc.

[28] Graham JW, Donaldson SI (1993). Evaluating interventions with differential attrition: the importance of nonresponse mechanisms and use of follow-up data. J Appl Psychol.

[29] Groenwold RH, Donders AR, Roes KC, Harrell FE, Moons KG (2012). Dealing with missing outcome data in randomized trials and observational studies. Am J Epidemiol.

[30] White IR, Carlin JB (2010). Bias and efficiency of multiple imputation compared with complete-case analysis for missing covariate values. Stat Med.

[31] Seaman SR, White IR (2013). Review of inverse probability weighting for dealing with missing data. Stat Methods Med Res.

