# Supplementary material for: Multiple imputation for handling missing outcome data when estimating the relative risk
Source: BMC Med Res Methodol. 2017 Sep 6;17:134. doi: 10.1186/s12874-017-0414-5 (PMC5588607; doi:10.1186/s12874-017-0414-5)
Supplement: Additional file 1: — Web Appendix. (DOCX 227 kb) [file 12874_2017_414_MOESM1_ESM.docx]

**Web Appendix**

SAS code for generating analysis datasets for simulation study 1: categorical exposures.

%macro categorical(seed, mvni_seed, fcs_seed, rrx, rry, intercept, mechanism);

*see note 1 for macro variable definitions;

*1. Generate x1, x2 and y;

data temp;

length simulation id 5.;

do simulation = 1 to 2000; *number of simulations = 2000;

do id = 1 to 1000; *sample size = 1000;

output;

end;

end;

run;

data temp;

set temp;

uniform1 = ranuni(&seed.);

uniform2 = ranuni(&seed.);

uniform3 = ranuni(&seed.);

uniform4 = ranuni(&seed.);

uniform5 = ranuni(&seed.);

run;

data temp;

set temp;

if uniform1 < 0.5 then x1 = 0;

else x1 = 1;

run;

%if &rrx. = 2 %then %do;

data temp;

set temp;

prob_x2 = (1/3) + (1/3)*x1;

run;

%end;

%else %if &rrx. = 3 %then %do;

data temp;

set temp;

prob_x2 = (1/4) + (2/4)*x1;

run;

%end;

data temp;

set temp;

if uniform2 < prob_x2 then x2 = 1;

else x2 = 0;

run;

data temp;

set temp;

prob_y = exp(&intercept. + log(&rry.)*x1 + log(&rry.)*x2);

*solve intercept computationally to give desired outcome prevalence, see note 2 after macro;

run;

data temp;

set temp;

if uniform3 <= prob_y then y = 1;

else y = 0;

run;

*2. Induce missing data in y and x2;

data temp;

set temp;

prob_y_strong = exp(-2.05 + 2*x1)/(1 + exp(-2.05 + 2*x1));*intercepts produce 30% missing data in y and 2;

prob_y_mod = exp(-1.40 + 1*x1)/(1 + exp(-1.40 + 1*x1));

prob_x2_strong = exp(-2.05 + 2*x1)/(1 + exp(-2.05 + 2*x1));

prob_x2_mod = exp(-1.40 + 1*x1)/(1 + exp(-1.40 + 1*x1));

prob_x2_opp_strong = exp(-0.05 - 2*x1)/(1 + exp(-0.05 - 2*x1));

prob_x2_opp_mod = exp(-0.40 - 1*x1)/(1 + exp(-0.40 - 1*x1));

run;

%if &mechanism. = "Coordinated_strong" %then %do;

data temp;

set temp;

if uniform4 <= prob_y_strong then missing_y = 1;

else missing_y = 0;

if uniform5 <= prob_x2_strong then missing_x2 = 1;

else missing_x2 = 0;

run;

%end;

%else %if &mechanism. = "Coordinated_mod" %then %do;

data temp;

set temp;

if uniform4 <= prob_y_mod then missing_y = 1;

else missing_y = 0;

if uniform5 <= prob_x2_mod then missing_x2 = 1;

else missing_x2 = 0;

run;

%end;

%else %if &mechanism. = "Opposite_strong" %then %do;

data temp;

set temp;

if uniform4 <= prob_y_strong then missing_y = 1;

else missing_y = 0;

if uniform5 <= prob_x2_opp_strong then missing_x2 = 1;

else missing_x2 = 0;

run;

%end;

%else %if &mechanism. = "Opposite_mod" %then %do;

data temp;

set temp;

if uniform4 <= prob_y_mod then missing_y = 1;

else missing_y = 0;

if uniform5 <= prob_x2_opp_mod then missing_x2 = 1;

else missing_x2 = 0;

run;

%end;

data temp;

set temp;

observed_y = y;

if missing_y = 1 then observed_y = .;

observed_x2 = x2;

if missing_x2 = 1 then observed_x2 = .;

keep simulation id x1 x2 y missing_y missing_x2 observed_y observed_x2;

run;

*3. Impute data;

*MVNI with adaptive rounding;

proc mi data=temp seed=&mvni_seed. nimpute=20 out=mvni;

by simulation;

mcmc chain=single initial=em;

var observed_y observed_x2 x1;

run;

proc univariate data = mvni;

by simulation _imputation_;

var observed_y observed_x2;

ods output BasicMeasures = bm;

run;

data temp1;

set bm;

where varname = 'observed_y' and locmeasure = 'Mean';

rename locvalue = w_y;

keep simulation _imputation_ locvalue;

run;

data temp2;

set bm;

where varname = 'observed_x2' and locmeasure = 'Mean';

rename locvalue = w_x2;

keep simulation _imputation_ locvalue;

run;

data mvni;

merge mvni temp1 temp2;

by simulation _imputation_;

threshold_y = w_y - (quantile('NORMAL', w_y)*sqrt(w_y*(1-w_y)));

threshold_x2 = w_x2 - (quantile('NORMAL', w_x2)*sqrt(w_x2*(1-w_x2)));

run;

data mvni;

set mvni;

if observed_y > threshold_y then observed_y = 1;

else if observed_y <= threshold_y then observed_y = 0;

if observed_x2 > threshold_x2 then observed_x2 = 1;

else if observed_x2 <= threshold_x2 then observed_x2 = 0;

run;

*FCS;

proc mi data=temp seed=&fcs_seed. nimpute=20 out=fcs;

by simulation;

class observed_x2 observed_y x1;

fcs logistic(observed_x2) logistic(observed_y) logistic(x1);

var observed_x2 observed_y x1;

run;

%mend;

/*

Note 1: macro variable definitions

seed: starting seed for random number generation

mvni_seed: starting seed for multivariate normal imputation

fcs_seed: starting seed for fully conditional specification

rrx: relative risk for association between X1 and X2

rry: relative risk for association between Y and X1, and Y and X2

intercept: intercept value for generating P(Y=1) (see note 2 below)

mechanism: missing data mechanism, takes values "Coordinated_strong", "Coordinated_mod", "Opposite_strong", "Opposite_mod" (see article for mechanism definitions)

Note 2: values for macro variable <intercept> used in simulation study:

rry = 2, rrx = 2, prevalence = 0.10, intercept = -3.15

rry = 2, rrx = 3, prevalence = 0.10, intercept = -3.17

rry = 2, rrx = 2, prevalence = 0.30, intercept = -2.05

rry = 2, rrx = 3, prevalence = 0.30, intercept = -2.07

rry = 3, rrx = 2, prevalence = 0.10, intercept = -3.77

rry = 3, rrx = 3, prevalence = 0.10, intercept = -3.81

rry = 3, rrx = 2, prevalence = 0.30, intercept = -2.67

rry = 3, rrx = 3, prevalence = 0.30, intercept = -2.71

*/

*Example call of macro for rrx = 2, rry = 3, prevalence = 0.30, strong coordinated mechanism;

%*categorical*(seed=1501, mvni_seed=1502, fcs_seed=1503, rrx=2, rry=3, intercept=-2.67, mechanism = "Coordinated_strong");

*Example analysis with MVNI;

proc genmod data=mvni descending;

class observed_y;

by simulation _imputation_;

model observed_y = observed_x2 x1 / link=log dist=binomial type3 wald covb;

ods output parameterestimates = _estimates covb = _cov convergenceStatus = cs1; *check convergence status;

run;

data _cov;

length parameter $11;

set _cov;

if rowname = 'Prm1' then Parameter = 'Intercept';

if rowname = 'Prm2' then Parameter = 'observed_x2';

if rowname = 'Prm3' then Parameter = 'x1';

drop rowname;

rename prm1 = Intercept prm2 = observed_x2 prm3 = x1;

run;

proc mianalyze parms=_estimates covb=_cov;

by simulation;

modeleffects Intercept observed_x2 x1;

ods output parameterestimates=est1;

run;

SAS code for generating analysis datasets for simulation study 2: continuous exposures.

%macro continuous(seed, mvni_seed, fcs_seed, corrx, rry, intercept, intercept_miss_y, intercept_miss_x2, mechanism);

*see note 1 for macro variable definitions;

*1. Generate x1, x2 and y;

data temp;

length simulation id 5.;

do simulation = 1 to 2000; *number of simulations = 2000;

do id = 1 to 1200;

*sample size = 1000, allowing extra observations so that out of range values for P(Y=1) can be replaced;

output;

end;

end;

run;

data temp;

set temp;

uniform1 = ranuni(&seed.);

uniform2 = ranuni(&seed.);

uniform3 = ranuni(&seed.);

normal1 = rannor(&seed.);

normal2 = rannor(&seed.);

run;

data temp;

set temp;

x1 = sqrt(0.2)*normal1;

run;

data temp;

set temp;

x2 = &corrx.*x1 + sqrt(0.2*(1-&corrx.*&corrx.))*normal2;

x1_std = x1/sqrt(0.2);

run;

data temp;

set temp;

prob_y = exp(&intercept. + log(&rry.)*x1 + log(&rry.)*x2);

*solve intercept computationally to give desired outcome prevalence, see note 2 after macro;

run;

data out_of_range;

set temp;

where prob_y > 1;

run;

data out_of_range;

set out_of_range;

count = _n_;

indicator = 1;

run;

data out_of_range;

set out_of_range;

by indicator;

if last.indicator;

percent_out_range = 100*count/(1200*2000); *calculate percentage simulated values outside of range;

keep percent_out_range;

run;

data temp;

set temp;

if prob_y > 1 then delete; *exclude observations where probability out of range;

run;

data temp;

retain counter;

set temp;

by simulation;

if first.simulation then counter = 1;

else counter = counter + 1;

run;

data temp;

set temp;

where counter <= 1000;

if uniform1 <= prob_y then y = 1;

else y = 0;

run;

*2. Induce missing data in y and x2;

%if &mechanism. = "Coordinated" %then %do;

data temp;

set temp;

prob_y_miss = exp(&intercept_miss_y. + 2*x1_std)/(1 + exp(&intercept_miss_y. + 2*x1_std));

prob_x2_miss = exp(&intercept_miss_x2. + 2*x1_std)/(1 + exp(&intercept_miss_x2. + 2*x1_std));

*solve intercept computationally to produce 30% missing data in y and x2, see note 3 after macro;

run;

%end;

%if &mechanism. = "Coordinated_mod" %then %do;

data temp;

set temp;

prob_y_miss = exp(&intercept_miss_y. + 1*x1_std)/(1 + exp(&intercept_miss_y. + 1*x1_std));

prob_x2_miss = exp(&intercept_miss_x2. + 1*x1_std)/(1 + exp(&intercept_miss_x2. + 1*x1_std));

run;

%end;

%if &mechanism. = "Opposite" %then %do;

data temp;

set temp;

prob_y_miss = exp(&intercept_miss_y. + 2*x1_std)/(1 + exp(&intercept_miss_y. + 2*x1_std));

prob_x2_miss = exp(&intercept_miss_x2. - 2*x1_std)/(1 + exp(&intercept_miss_x2. - 2*x1_std));

run;

%end;

%if &mechanism. = "Opposite_mod" %then %do;

data temp;

set temp;

prob_y_miss = exp(&intercept_miss_y. + 1*x1_std)/(1 + exp(&intercept_miss_y. + 1*x1_std));

prob_x2_miss = exp(&intercept_miss_x2. - 1*x1_std)/(1 + exp(&intercept_miss_x2. - 1*x1_std));

run;

%end;

data temp;

set temp;

if uniform2 <= prob_y_miss then missing_y = 1;

else missing_y = 0;

if uniform3 <= prob_x2_miss then missing_x2 = 1;

else missing_x2 = 0;

run;

data temp;

set temp;

observed_y = y;

if missing_y = 1 then observed_y = .;

observed_x2 = x2;

if missing_x2 = 1 then observed_x2 = .;

keep simulation id x1 x2 y missing_y missing_x2 observed_y observed_x2;

run;

*3. Impute data;

*MVNI with adaptive rounding;

proc mi data=temp seed=&mvni_seed. nimpute=20 out=mvni;

by simulation;

mcmc chain=single initial=em;

var observed_y observed_x2 x1;

run;

proc univariate data = mvni;

by simulation _imputation_;

var observed_y;

ods output BasicMeasures = bm;

run;

data temp1;

set bm;

where varname = 'observed_y' and locmeasure = 'Mean';

rename locvalue = w_y;

keep simulation _imputation_ locvalue;

run;

data mvni;

merge mvni temp1;

by simulation _imputation_;

threshold_y = w_y - (quantile('NORMAL', w_y)*sqrt(w_y*(1-w_y)));

run;

data mvni;

set mvni;

if observed_y > threshold_y then observed_y = 1;

else if observed_y <= threshold_y then observed_y = 0;

run;

*FCS;

proc mi data=temp seed=&fcs_seed. nimpute=20 out=fcs;

by simulation;

class observed_y;

fcs reg(observed_x2) logistic(observed_y) reg(x1);

var observed_x2 observed_y x1;

run;

%mend;

/*

Note 1: macro variable definitions

seed: starting seed for random number generation

mvni_seed: starting seed for multivariate normal imputation

fcs_seed: starting seed for fully conditional specification

corrx: correlation for association between X1 and X2

rry: relative risk for association between Y and X1, and Y and X2

intercept: intercept value for generating P(Y=1) (see note 2 below)

intercept_miss_y: intercept value for generating 30% missing data in Y (see note 3 below)

intercept_miss_x2: intercept value for generating 30% missing data in X2 (see note 3 below)

mechanism: missing data mechanism, takes values "Coordinated_strong", "Coordinated_mod", "Opposite_strong", "Opposite_mod" (see article for mechanism definitions)

Note 2: values for macro variable <intercept> used in simulation study:

rry = 2, corrx = 0.30, prevalence = 0.10, intercept = -2.43

rry = 2, corrx = 0.70, prevalence = 0.10, intercept = -2.46

rry = 2, corrx = 0.30, prevalence = 0.30, intercept = -1.32

rry = 2, corrx = 0.70, prevalence = 0.30, intercept = -1.34

rry = 3, corrx = 0.30, prevalence = 0.10, intercept = -2.61

rry = 3, corrx = 0.70, prevalence = 0.10, intercept = -2.69

rry = 3, corrx = 0.30, prevalence = 0.30, intercept = -1.37

rry = 3, corrx = 0.70, prevalence = 0.30, intercept = -1.36

Note 3: values for macro variables <intercept_miss_y> and <intercept_miss_x2> used in simulation study:

Moderate mechanism, intercept_miss_y = intercept_miss_x2 = -1.02

Strong mechanism, intercept_miss_y = intercept_miss_x2 = -1.39

-> Note intercept values adjusted in scenarios with an outcome prevalence of 0.30 to maintain 30% missing data after replacing out of range values for P(Y=1)

*/

*Example call of macro for corrx = 0.70, rry = 2, prevalence = 0.10, moderate coordinated mechanism;

%*continuous* (seed=12601, mvni_seed=12602, fcs_seed=12603, corrx=0.70, rry=2, intercept=-2.46, intercept_miss_y = -1.02,

intercept_miss_x2 = -1.02, mechanism = "Coordinated_mod");

*Example analysis with FCS (using log Poisson GEE to estimate the relative risk);

proc genmod data=fcs;

class id;

by simulation _imputation_;

model observed_y = observed_x2 x1 / link=log dist=poisson type3 wald covb;

repeated subject = id /type=ind;

ods output GEEemppest = _estimates covb = _cov;

run;

data _estimates;

set _estimates;

rename parm = parameter;

keep simulation _imputation_ Parm Estimate stderr;

run;

data _cov;

length parameter $11;

set _cov;

if rowname = 'Prm1' then Parameter = 'Intercept';

if rowname = 'Prm2' then Parameter = 'observed_x2';

if rowname = 'Prm3' then Parameter = 'x1';

drop rowname;

rename prm1 = Intercept prm2 = observed_x2 prm3 = x1;

run;

proc mianalyze parms=_estimates covb=_cov;

by simulation;

modeleffects Intercept observed_x2 x1;

ods output parameterestimates=est3;

run;

**Web Table 1** Full Simulation Results for $X_{1}$ and $X_{2}$ Binary

| Missing data mechanism | $\lambda$^a^ | Outcome prevalence | $\beta_{1},\beta_{2}$ | $\mathrm{RR}\left( X_{1},X_{2} \right)$ | Method | $\beta_{1}$ | | | | | $\beta_{2}$ | | | | |
| --- | --- | --- | --- | --- | --- | --- | --- | --- | --- | --- | --- | --- | --- | --- | --- |
|  |  |  |  |  |  | Bias | Avg SE | Emp SE | Coverage | MSE | Bias | Avg SE | Emp SE | Coverage | MSE |
| Coordinated | 1 | 0.10 | Log(2) | 2 | MVNI | -0.04 | 0.26 | 0.24 | 0.961 | 0.06 | -0.14 | 0.30 | 0.26 | 0.941 | 0.09 |
|  |  |  |  |  | MVNI + deletion | 0.05 | 0.27 | 0.27 | 0.948 | 0.08 | -0.05 | 0.32 | 0.30 | 0.960 | 0.10 |
|  |  |  |  |  | FCS | 0.02 | 0.27 | 0.28 | 0.945 | 0.08 | 0.00 | 0.33 | 0.34 | 0.957 | 0.11 |
|  |  |  |  |  | FCS + deletion | 0.02 | 0.27 | 0.28 | 0.947 | 0.08 | 0.01 | 0.33 | 0.34 | 0.957 | 0.11 |
|  |  |  |  |  | CCA | 0.02 | 0.31 | 0.32 | 0.954 | 0.10 | 0.01 | 0.33 | 0.33 | 0.957 | 0.11 |
| Coordinated | 1 | 0.10 | Log(2) | 3 | MVNI | -0.04 | 0.29 | 0.27 | 0.968 | 0.07 | -0.14 | 0.33 | 0.28 | 0.957 | 0.10 |
|  |  |  |  |  | MVNI + deletion | 0.05 | 0.30 | 0.30 | 0.958 | 0.09 | -0.05 | 0.36 | 0.33 | 0.962 | 0.11 |
|  |  |  |  |  | FCS | 0.02 | 0.31 | 0.31 | 0.955 | 0.10 | 0.01 | 0.37 | 0.37 | 0.947 | 0.14 |
|  |  |  |  |  | FCS + deletion | 0.01 | 0.30 | 0.31 | 0.952 | 0.10 | 0.02 | 0.37 | 0.37 | 0.951 | 0.14 |
|  |  |  |  |  | CCA | 0.00 | 0.34 | 0.34 | 0.953 | 0.12 | 0.03 | 0.36 | 0.37 | 0.948 | 0.14 |
| Coordinated | 1 | 0.10 | Log(3) | 2 | MVNI | -0.07 | 0.30 | 0.27 | 0.958 | 0.08 | -0.27 | 0.34 | 0.27 | 0.895 | 0.15 |
|  |  |  |  |  | MVNI + deletion | 0.08 | 0.30 | 0.32 | 0.951 | 0.11 | -0.10 | 0.38 | 0.35 | 0.944 | 0.13 |
|  |  |  |  |  | FCS | 0.04 | 0.31 | 0.32 | 0.953 | 0.10 | 0.02 | 0.40 | 0.41 | 0.954 | 0.17 |
|  |  |  |  |  | FCS + deletion | 0.03 | 0.30 | 0.32 | 0.953 | 0.10 | 0.03 | 0.40 | 0.41 | 0.958 | 0.17 |
|  |  |  |  |  | CCA | 0.04 | 0.35 | 0.36 | 0.953 | 0.13 | 0.03 | 0.39 | 0.41 | 0.951 | 0.17 |
| Coordinated | 1 | 0.10 | Log(3) | 3 | MVNI | -0.04 | 0.34 | 0.30 | 0.969 | 0.09 | -0.29 | 0.37 | 0.30 | 0.901 | 0.17 |
|  |  |  |  |  | MVNI + deletion | 0.11 | 0.35 | 0.35 | 0.957 | 0.13 | -0.11 | 0.42 | 0.38 | 0.954 | 0.16 |
|  |  |  |  |  | FCS | 0.05 | 0.35 | 0.35 | 0.955 | 0.13 | 0.02 | 0.44 | 0.45 | 0.957 | 0.21 |
|  |  |  |  |  | FCS + deletion | 0.05 | 0.35 | 0.35 | 0.948 | 0.13 | 0.03 | 0.44 | 0.45 | 0.958 | 0.21 |
|  |  |  |  |  | CCA | 0.04 | 0.39 | 0.39 | 0.954 | 0.16 | 0.04 | 0.44 | 0.45 | 0.959 | 0.21 |
| Coordinated | 1 | 0.30 | Log(2) | 2 | MVNI | -0.01 | 0.14 | 0.13 | 0.959 | 0.02 | -0.13 | 0.15 | 0.14 | 0.884 | 0.04 |
|  |  |  |  |  | MVNI + deletion | 0.03 | 0.14 | 0.14 | 0.950 | 0.02 | -0.07 | 0.17 | 0.15 | 0.941 | 0.03 |
|  |  |  |  |  | FCS | 0.01 | 0.14 | 0.14 | 0.949 | 0.02 | -0.03 | 0.17 | 0.17 | 0.954 | 0.03 |
|  |  |  |  |  | FCS + deletion | 0.01 | 0.14 | 0.14 | 0.950 | 0.02 | -0.01 | 0.17 | 0.17 | 0.953 | 0.03 |
|  |  |  |  |  | CCA | 0.01 | 0.16 | 0.16 | 0.949 | 0.02 | 0.00 | 0.17 | 0.17 | 0.957 | 0.03 |
| Coordinated | 1 | 0.30 | Log(2) | 3 | MVNI | 0.01 | 0.15 | 0.15 | 0.958 | 0.02 | -0.14 | 0.17 | 0.16 | 0.881 | 0.04 |
|  |  |  |  |  | MVNI + deletion | 0.05 | 0.16 | 0.15 | 0.949 | 0.03 | -0.07 | 0.18 | 0.17 | 0.939 | 0.03 |
|  |  |  |  |  | FCS | 0.02 | 0.16 | 0.16 | 0.953 | 0.02 | -0.02 | 0.19 | 0.19 | 0.950 | 0.04 |
|  |  |  |  |  | FCS + deletion | 0.01 | 0.16 | 0.16 | 0.949 | 0.02 | -0.01 | 0.19 | 0.19 | 0.952 | 0.04 |
|  |  |  |  |  | CCA | 0.00 | 0.17 | 0.17 | 0.948 | 0.03 | 0.01 | 0.19 | 0.19 | 0.951 | 0.04 |
| Coordinated | 1 | 0.30 | Log(3) | 2 | MVNI | -0.01 | 0.16 | 0.15 | 0.955 | 0.02 | -0.26 | 0.17 | 0.15 | 0.682 | 0.09 |
|  |  |  |  |  | MVNI + deletion | 0.05 | 0.16 | 0.16 | 0.948 | 0.03 | -0.12 | 0.19 | 0.18 | 0.893 | 0.05 |
|  |  |  |  |  | FCS | 0.01 | 0.16 | 0.16 | 0.954 | 0.03 | -0.06 | 0.20 | 0.20 | 0.933 | 0.04 |
|  |  |  |  |  | FCS + deletion | 0.01 | 0.16 | 0.16 | 0.951 | 0.03 | -0.03 | 0.20 | 0.20 | 0.946 | 0.04 |
|  |  |  |  |  | CCA | 0.01 | 0.18 | 0.18 | 0.954 | 0.03 | 0.01 | 0.20 | 0.20 | 0.955 | 0.04 |
| Coordinated | 1 | 0.30 | Log(3) | 3 | MVNI | 0.03 | 0.18 | 0.18 | 0.948 | 0.03 | -0.27 | 0.19 | 0.18 | 0.719 | 0.10 |
|  |  |  |  |  | MVNI + deletion | 0.08 | 0.18 | 0.19 | 0.931 | 0.04 | -0.13 | 0.22 | 0.20 | 0.901 | 0.06 |
|  |  |  |  |  | FCS | 0.03 | 0.19 | 0.19 | 0.940 | 0.04 | -0.06 | 0.23 | 0.22 | 0.934 | 0.05 |
|  |  |  |  |  | FCS + deletion | 0.02 | 0.18 | 0.19 | 0.938 | 0.04 | -0.02 | 0.23 | 0.23 | 0.945 | 0.05 |
|  |  |  |  |  | CCA | 0.01 | 0.20 | 0.21 | 0.942 | 0.04 | 0.01 | 0.23 | 0.23 | 0.950 | 0.05 |
| Coordinated | 2 | 0.10 | Log(2) | 2 | MVNI | -0.07 | 0.26 | 0.24 | 0.954 | 0.06 | -0.15 | 0.30 | 0.26 | 0.936 | 0.09 |
|  |  |  |  |  | MVNI + deletion | 0.03 | 0.27 | 0.27 | 0.956 | 0.07 | -0.05 | 0.33 | 0.31 | 0.958 | 0.10 |
|  |  |  |  |  | FCS | 0.01 | 0.27 | 0.27 | 0.947 | 0.07 | -0.00 | 0.34 | 0.34 | 0.957 | 0.12 |
|  |  |  |  |  | FCS + deletion | 0.01 | 0.27 | 0.27 | 0.950 | 0.07 | 0.00 | 0.34 | 0.34 | 0.955 | 0.12 |
|  |  |  |  |  | CCA | -0.01 | 0.32 | 0.32 | 0.954 | 0.10 | 0.01 | 0.34 | 0.34 | 0.953 | 0.11 |
| Coordinated | 2 | 0.10 | Log(2) | 3 | MVNI | -0.05 | 0.29 | 0.28 | 0.951 | 0.08 | -0.14 | 0.33 | 0.29 | 0.948 | 0.11 |
|  |  |  |  |  | MVNI + deletion | 0.05 | 0.30 | 0.31 | 0.947 | 0.10 | -0.04 | 0.36 | 0.34 | 0.956 | 0.12 |
|  |  |  |  |  | FCS | 0.02 | 0.30 | 0.31 | 0.942 | 0.10 | 0.00 | 0.37 | 0.37 | 0.949 | 0.14 |
|  |  |  |  |  | FCS + deletion | 0.01 | 0.30 | 0.31 | 0.943 | 0.10 | 0.01 | 0.37 | 0.37 | 0.950 | 0.14 |
|  |  |  |  |  | CCA | -0.01 | 0.35 | 0.36 | 0.952 | 0.13 | 0.02 | 0.36 | 0.37 | 0.948 | 0.14 |
| Coordinated | 2 | 0.10 | Log(3) | 2 | MVNI | -0.08 | 0.30 | 0.28 | 0.951 | 0.08 | -0.28 | 0.35 | 0.28 | 0.896 | 0.15 |
|  |  |  |  |  | MVNI + deletion | 0.06 | 0.30 | 0.31 | 0.955 | 0.10 | -0.09 | 0.39 | 0.35 | 0.956 | 0.13 |
|  |  |  |  |  | FCS | 0.02 | 0.30 | 0.31 | 0.955 | 0.10 | -0.00 | 0.40 | 0.40 | 0.962 | 0.16 |
|  |  |  |  |  | FCS + deletion | 0.02 | 0.30 | 0.31 | 0.948 | 0.09 | 0.01 | 0.40 | 0.40 | 0.962 | 0.16 |
|  |  |  |  |  | CCA | 0.01 | 0.34 | 0.34 | 0.953 | 0.12 | 0.03 | 0.40 | 0.40 | 0.964 | 0.16 |
| Coordinated | 2 | 0.10 | Log(3) | 3 | MVNI | -0.03 | 0.34 | 0.32 | 0.961 | 0.10 | -0.28 | 0.39 | 0.32 | 0.893 | 0.18 |
|  |  |  |  |  | MVNI + deletion | 0.10 | 0.34 | 0.35 | 0.955 | 0.13 | -0.10 | 0.43 | 0.39 | 0.955 | 0.16 |
|  |  |  |  |  | FCS | 0.05 | 0.35 | 0.35 | 0.947 | 0.13 | -0.00 | 0.44 | 0.45 | 0.956 | 0.20 |
|  |  |  |  |  | FCS + deletion | 0.04 | 0.34 | 0.35 | 0.949 | 0.12 | 0.02 | 0.44 | 0.45 | 0.960 | 0.20 |
|  |  |  |  |  | CCA | 0.03 | 0.38 | 0.40 | 0.951 | 0.16 | 0.04 | 0.44 | 0.45 | 0.963 | 0.20 |
| Coordinated | 2 | 0.30 | Log(2) | 2 | MVNI | 0.00 | 0.14 | 0.14 | 0.949 | 0.02 | -0.16 | 0.16 | 0.14 | 0.836 | 0.04 |
|  |  |  |  |  | MVNI + deletion | 0.03 | 0.14 | 0.14 | 0.939 | 0.02 | -0.07 | 0.17 | 0.16 | 0.933 | 0.03 |
|  |  |  |  |  | FCS | 0.01 | 0.14 | 0.14 | 0.943 | 0.02 | -0.05 | 0.17 | 0.17 | 0.940 | 0.03 |
|  |  |  |  |  | FCS + deletion | 0.01 | 0.14 | 0.14 | 0.946 | 0.02 | -0.02 | 0.17 | 0.17 | 0.949 | 0.03 |
|  |  |  |  |  | CCA | -0.00 | 0.16 | 0.16 | 0.945 | 0.03 | 0.00 | 0.17 | 0.17 | 0.953 | 0.03 |
| Coordinated | 2 | 0.30 | Log(2) | 3 | MVNI | 0.03 | 0.15 | 0.15 | 0.955 | 0.02 | -0.16 | 0.17 | 0.16 | 0.843 | 0.05 |
|  |  |  |  |  | MVNI + deletion | 0.05 | 0.15 | 0.15 | 0.949 | 0.03 | -0.07 | 0.18 | 0.18 | 0.927 | 0.04 |
|  |  |  |  |  | FCS | 0.02 | 0.16 | 0.16 | 0.948 | 0.03 | -0.05 | 0.19 | 0.19 | 0.930 | 0.04 |
|  |  |  |  |  | FCS + deletion | 0.01 | 0.15 | 0.16 | 0.945 | 0.02 | -0.02 | 0.19 | 0.19 | 0.940 | 0.04 |
|  |  |  |  |  | CCA | 0.00 | 0.17 | 0.17 | 0.954 | 0.03 | 0.00 | 0.19 | 0.20 | 0.941 | 0.04 |
| Coordinated | 2 | 0.30 | Log(3) | 2 | MVNI | 0.03 | 0.16 | 0.15 | 0.952 | 0.02 | -0.32 | 0.17 | 0.16 | 0.547 | 0.13 |
|  |  |  |  |  | MVNI + deletion | 0.05 | 0.16 | 0.16 | 0.948 | 0.03 | -0.15 | 0.20 | 0.19 | 0.872 | 0.06 |
|  |  |  |  |  | FCS | 0.03 | 0.16 | 0.16 | 0.951 | 0.03 | -0.11 | 0.20 | 0.21 | 0.893 | 0.05 |
|  |  |  |  |  | FCS + deletion | 0.02 | 0.16 | 0.16 | 0.955 | 0.02 | -0.06 | 0.21 | 0.21 | 0.932 | 0.05 |
|  |  |  |  |  | CCA | 0.01 | 0.17 | 0.17 | 0.953 | 0.03 | 0.01 | 0.21 | 0.22 | 0.949 | 0.05 |
| Coordinated | 2 | 0.30 | Log(3) | 3 | MVNI | 0.07 | 0.18 | 0.18 | 0.938 | 0.04 | -0.31 | 0.20 | 0.18 | 0.640 | 0.13 |
|  |  |  |  |  | MVNI + deletion | 0.07 | 0.18 | 0.18 | 0.939 | 0.04 | -0.14 | 0.22 | 0.21 | 0.895 | 0.06 |
|  |  |  |  |  | FCS | 0.04 | 0.18 | 0.18 | 0.946 | 0.04 | -0.10 | 0.23 | 0.22 | 0.921 | 0.06 |
|  |  |  |  |  | FCS + deletion | 0.02 | 0.18 | 0.18 | 0.949 | 0.03 | -0.04 | 0.23 | 0.23 | 0.939 | 0.06 |
|  |  |  |  |  | CCA | -0.00 | 0.19 | 0.20 | 0.952 | 0.04 | 0.02 | 0.23 | 0.23 | 0.942 | 0.06 |
| Opposite | 1 | 0.10 | Log(2) | 2 | MVNI | -0.05 | 0.26 | 0.23 | 0.964 | 0.06 | -0.13 | 0.30 | 0.25 | 0.953 | 0.08 |
|  |  |  |  |  | MVNI + deletion | 0.04 | 0.27 | 0.26 | 0.958 | 0.07 | -0.03 | 0.32 | 0.30 | 0.961 | 0.09 |
|  |  |  |  |  | FCS | 0.01 | 0.27 | 0.27 | 0.953 | 0.07 | 0.03 | 0.33 | 0.34 | 0.950 | 0.12 |
|  |  |  |  |  | FCS + deletion | 0.01 | 0.27 | 0.27 | 0.954 | 0.07 | 0.03 | 0.33 | 0.34 | 0.950 | 0.12 |
|  |  |  |  |  | CCA | 0.01 | 0.33 | 0.34 | 0.950 | 0.11 | 0.03 | 0.33 | 0.34 | 0.951 | 0.12 |
| Opposite | 1 | 0.10 | Log(2) | 3 | MVNI | -0.04 | 0.29 | 0.26 | 0.969 | 0.07 | -0.14 | 0.32 | 0.28 | 0.950 | 0.10 |
|  |  |  |  |  | MVNI + deletion | 0.05 | 0.30 | 0.29 | 0.961 | 0.09 | -0.04 | 0.35 | 0.33 | 0.957 | 0.11 |
|  |  |  |  |  | FCS | 0.00 | 0.31 | 0.30 | 0.951 | 0.09 | 0.04 | 0.37 | 0.38 | 0.949 | 0.15 |
|  |  |  |  |  | FCS + deletion | -0.00 | 0.30 | 0.30 | 0.951 | 0.09 | 0.04 | 0.36 | 0.38 | 0.946 | 0.15 |
|  |  |  |  |  | CCA | 0.01 | 0.36 | 0.37 | 0.950 | 0.14 | 0.04 | 0.36 | 0.38 | 0.947 | 0.14 |
| Opposite | 1 | 0.10 | Log(3) | 2 | MVNI | -0.08 | 0.30 | 0.26 | 0.956 | 0.08 | -0.24 | 0.33 | 0.26 | 0.907 | 0.13 |
|  |  |  |  |  | MVNI + deletion | 0.07 | 0.30 | 0.31 | 0.952 | 0.10 | -0.06 | 0.37 | 0.34 | 0.959 | 0.12 |
|  |  |  |  |  | FCS | 0.02 | 0.31 | 0.32 | 0.948 | 0.10 | 0.06 | 0.39 | 0.41 | 0.953 | 0.17 |
|  |  |  |  |  | FCS + deletion | 0.02 | 0.30 | 0.31 | 0.946 | 0.10 | 0.07 | 0.39 | 0.41 | 0.953 | 0.17 |
|  |  |  |  |  | CCA | 0.06 | 0.38 | 0.69 | 0.950 | 0.48 | 0.06 | 0.38 | 0.41 | 0.952 | 0.17 |
| Opposite | 1 | 0.10 | Log(3) | 3 | MVNI | -0.04 | 0.33 | 0.29 | 0.973 | 0.09 | -0.27 | 0.36 | 0.28 | 0.907 | 0.15 |
|  |  |  |  |  | MVNI + deletion | 0.10 | 0.35 | 0.35 | 0.955 | 0.13 | -0.09 | 0.40 | 0.36 | 0.957 | 0.14 |
|  |  |  |  |  | FCS | 0.03 | 0.35 | 0.36 | 0.948 | 0.13 | 0.05 | 0.43 | 0.45 | 0.955 | 0.21 |
|  |  |  |  |  | FCS + deletion | 0.03 | 0.35 | 0.36 | 0.945 | 0.13 | 0.06 | 0.43 | 0.45 | 0.958 | 0.21 |
|  |  |  |  |  | CCA | 0.06 | 0.43 | 0.45 | 0.956 | 0.21 | 0.05 | 0.43 | 0.45 | 0.959 | 0.20 |
| Opposite | 1 | 0.30 | Log(2) | 2 | MVNI | -0.02 | 0.14 | 0.13 | 0.951 | 0.02 | -0.10 | 0.15 | 0.14 | 0.905 | 0.03 |
|  |  |  |  |  | MVNI + deletion | 0.02 | 0.14 | 0.14 | 0.945 | 0.02 | -0.03 | 0.16 | 0.16 | 0.943 | 0.03 |
|  |  |  |  |  | FCS | -0.00 | 0.14 | 0.14 | 0.946 | 0.02 | 0.00 | 0.17 | 0.17 | 0.947 | 0.03 |
|  |  |  |  |  | FCS + deletion | -0.00 | 0.14 | 0.14 | 0.946 | 0.02 | 0.02 | 0.17 | 0.17 | 0.944 | 0.03 |
|  |  |  |  |  | CCA | 0.01 | 0.17 | 0.17 | 0.949 | 0.03 | 0.01 | 0.17 | 0.17 | 0.944 | 0.03 |
| Opposite | 1 | 0.30 | Log(2) | 3 | MVNI | 0.00 | 0.15 | 0.14 | 0.964 | 0.02 | -0.11 | 0.17 | 0.15 | 0.901 | 0.04 |
|  |  |  |  |  | MVNI + deletion | 0.04 | 0.16 | 0.15 | 0.949 | 0.02 | -0.04 | 0.18 | 0.17 | 0.945 | 0.03 |
|  |  |  |  |  | FCS | 0.00 | 0.16 | 0.16 | 0.956 | 0.02 | 0.01 | 0.19 | 0.19 | 0.947 | 0.03 |
|  |  |  |  |  | FCS + deletion | -0.00 | 0.16 | 0.16 | 0.952 | 0.02 | 0.02 | 0.19 | 0.19 | 0.951 | 0.04 |
|  |  |  |  |  | CCA | 0.01 | 0.18 | 0.18 | 0.949 | 0.03 | 0.01 | 0.18 | 0.18 | 0.948 | 0.03 |
| Opposite | 1 | 0.30 | Log(3) | 2 | MVNI | -0.03 | 0.16 | 0.15 | 0.955 | 0.02 | -0.19 | 0.17 | 0.16 | 0.798 | 0.06 |
|  |  |  |  |  | MVNI + deletion | 0.03 | 0.16 | 0.15 | 0.958 | 0.02 | -0.05 | 0.19 | 0.18 | 0.942 | 0.04 |
|  |  |  |  |  | FCS | -0.01 | 0.16 | 0.16 | 0.952 | 0.02 | 0.00 | 0.20 | 0.20 | 0.954 | 0.04 |
|  |  |  |  |  | FCS + deletion | -0.01 | 0.16 | 0.16 | 0.947 | 0.02 | 0.04 | 0.20 | 0.20 | 0.952 | 0.04 |
|  |  |  |  |  | CCA | 0.00 | 0.19 | 0.20 | 0.949 | 0.04 | 0.01 | 0.19 | 0.20 | 0.955 | 0.04 |
| Opposite | 1 | 0.30 | Log(3) | 3 | MVNI | 0.01 | 0.18 | 0.17 | 0.960 | 0.03 | -0.21 | 0.19 | 0.18 | 0.810 | 0.07 |
|  |  |  |  |  | MVNI + deletion | 0.05 | 0.18 | 0.18 | 0.951 | 0.04 | -0.07 | 0.21 | 0.20 | 0.944 | 0.05 |
|  |  |  |  |  | FCS | -0.00 | 0.19 | 0.19 | 0.947 | 0.04 | 0.01 | 0.23 | 0.23 | 0.949 | 0.05 |
|  |  |  |  |  | FCS + deletion | -0.01 | 0.18 | 0.19 | 0.944 | 0.04 | 0.05 | 0.23 | 0.23 | 0.949 | 0.06 |
|  |  |  |  |  | CCA | 0.01 | 0.22 | 0.23 | 0.943 | 0.05 | 0.02 | 0.22 | 0.23 | 0.946 | 0.05 |
| Opposite | 2 | 0.10 | Log(2) | 2 | MVNI | -0.06 | 0.26 | 0.24 | 0.963 | 0.06 | -0.13 | 0.31 | 0.27 | 0.951 | 0.09 |
|  |  |  |  |  | MVNI + deletion | 0.04 | 0.27 | 0.27 | 0.959 | 0.07 | -0.03 | 0.33 | 0.32 | 0.960 | 0.10 |
|  |  |  |  |  | FCS | 0.01 | 0.27 | 0.27 | 0.959 | 0.07 | 0.03 | 0.34 | 0.36 | 0.955 | 0.13 |
|  |  |  |  |  | FCS + deletion | 0.01 | 0.27 | 0.27 | 0.959 | 0.07 | 0.03 | 0.34 | 0.36 | 0.946 | 0.13 |
|  |  |  |  |  | CCA | 0.02 | 0.34 | 0.34 | 0.962 | 0.12 | 0.02 | 0.34 | 0.36 | 0.954 | 0.13 |
| Opposite | 2 | 0.10 | Log(2) | 3 | MVNI | -0.05 | 0.29 | 0.27 | 0.959 | 0.08 | -0.14 | 0.33 | 0.28 | 0.952 | 0.10 |
|  |  |  |  |  | MVNI + deletion | 0.05 | 0.30 | 0.30 | 0.949 | 0.09 | -0.04 | 0.36 | 0.33 | 0.960 | 0.11 |
|  |  |  |  |  | FCS | 0.01 | 0.31 | 0.31 | 0.943 | 0.10 | 0.04 | 0.38 | 0.39 | 0.948 | 0.15 |
|  |  |  |  |  | FCS + deletion | -0.00 | 0.30 | 0.31 | 0.940 | 0.10 | 0.05 | 0.38 | 0.39 | 0.949 | 0.16 |
|  |  |  |  |  | CCA | 0.03 | 0.37 | 0.39 | 0.954 | 0.15 | 0.04 | 0.37 | 0.39 | 0.947 | 0.15 |
| Opposite | 2 | 0.10 | Log(3) | 2 | MVNI | -0.08 | 0.29 | 0.28 | 0.949 | 0.08 | -0.26 | 0.34 | 0.26 | 0.908 | 0.13 |
|  |  |  |  |  | MVNI + deletion | 0.05 | 0.30 | 0.30 | 0.955 | 0.09 | -0.07 | 0.37 | 0.33 | 0.964 | 0.11 |
|  |  |  |  |  | FCS | 0.01 | 0.30 | 0.31 | 0.952 | 0.10 | 0.03 | 0.39 | 0.39 | 0.963 | 0.16 |
|  |  |  |  |  | FCS + deletion | 0.01 | 0.30 | 0.31 | 0.950 | 0.09 | 0.05 | 0.39 | 0.40 | 0.964 | 0.16 |
|  |  |  |  |  | CCA | 0.03 | 0.39 | 0.41 | 0.956 | 0.17 | 0.03 | 0.39 | 0.39 | 0.965 | 0.15 |
| Opposite | 2 | 0.10 | Log(3) | 3 | MVNI | -0.04 | 0.33 | 0.30 | 0.967 | 0.09 | -0.27 | 0.38 | 0.29 | 0.906 | 0.16 |
|  |  |  |  |  | MVNI + deletion | 0.09 | 0.34 | 0.32 | 0.965 | 0.11 | -0.09 | 0.41 | 0.37 | 0.952 | 0.14 |
|  |  |  |  |  | FCS | 0.02 | 0.34 | 0.34 | 0.956 | 0.12 | 0.05 | 0.44 | 0.46 | 0.956 | 0.22 |
|  |  |  |  |  | FCS + deletion | 0.01 | 0.34 | 0.34 | 0.956 | 0.11 | 0.07 | 0.44 | 0.47 | 0.957 | 0.22 |
|  |  |  |  |  | CCA | 0.05 | 0.44 | 0.45 | 0.961 | 0.20 | 0.05 | 0.44 | 0.46 | 0.954 | 0.21 |
| Opposite | 2 | 0.30 | Log(2) | 2 | MVNI | -0.01 | 0.14 | 0.13 | 0.955 | 0.02 | -0.10 | 0.16 | 0.14 | 0.916 | 0.03 |
|  |  |  |  |  | MVNI + deletion | 0.02 | 0.14 | 0.14 | 0.952 | 0.02 | -0.02 | 0.17 | 0.16 | 0.959 | 0.03 |
|  |  |  |  |  | FCS | -0.01 | 0.14 | 0.14 | 0.949 | 0.02 | 0.01 | 0.17 | 0.17 | 0.953 | 0.03 |
|  |  |  |  |  | FCS + deletion | -0.01 | 0.14 | 0.14 | 0.946 | 0.02 | 0.03 | 0.18 | 0.18 | 0.955 | 0.03 |
|  |  |  |  |  | CCA | 0.00 | 0.17 | 0.18 | 0.951 | 0.03 | 0.01 | 0.17 | 0.17 | 0.952 | 0.03 |
| Opposite | 2 | 0.30 | Log(2) | 3 | MVNI | 0.01 | 0.15 | 0.15 | 0.952 | 0.02 | -0.11 | 0.17 | 0.16 | 0.914 | 0.04 |
|  |  |  |  |  | MVNI + deletion | 0.03 | 0.15 | 0.15 | 0.950 | 0.02 | -0.03 | 0.19 | 0.18 | 0.953 | 0.03 |
|  |  |  |  |  | FCS | -0.01 | 0.16 | 0.16 | 0.941 | 0.03 | 0.01 | 0.19 | 0.20 | 0.954 | 0.04 |
|  |  |  |  |  | FCS + deletion | -0.02 | 0.16 | 0.16 | 0.938 | 0.03 | 0.04 | 0.19 | 0.20 | 0.948 | 0.04 |
|  |  |  |  |  | CCA | 0.00 | 0.19 | 0.20 | 0.935 | 0.04 | 0.01 | 0.19 | 0.19 | 0.951 | 0.04 |
| Opposite | 2 | 0.30 | Log(3) | 2 | MVNI | -0.00 | 0.16 | 0.15 | 0.961 | 0.02 | -0.20 | 0.18 | 0.16 | 0.805 | 0.06 |
|  |  |  |  |  | MVNI + deletion | 0.03 | 0.16 | 0.15 | 0.961 | 0.02 | -0.02 | 0.20 | 0.19 | 0.952 | 0.03 |
|  |  |  |  |  | FCS | -0.00 | 0.16 | 0.16 | 0.951 | 0.02 | 0.01 | 0.20 | 0.20 | 0.948 | 0.04 |
|  |  |  |  |  | FCS + deletion | -0.02 | 0.16 | 0.15 | 0.949 | 0.02 | 0.07 | 0.21 | 0.21 | 0.947 | 0.05 |
|  |  |  |  |  | CCA | 0.01 | 0.20 | 0.20 | 0.952 | 0.04 | 0.02 | 0.20 | 0.20 | 0.952 | 0.04 |
| Opposite | 2 | 0.30 | Log(3) | 3 | MVNI | 0.04 | 0.18 | 0.17 | 0.961 | 0.03 | -0.22 | 0.20 | 0.18 | 0.809 | 0.08 |
|  |  |  |  |  | MVNI + deletion | 0.05 | 0.18 | 0.17 | 0.961 | 0.03 | -0.04 | 0.22 | 0.21 | 0.946 | 0.05 |
|  |  |  |  |  | FCS | -0.00 | 0.18 | 0.18 | 0.956 | 0.03 | 0.01 | 0.23 | 0.24 | 0.948 | 0.06 |
|  |  |  |  |  | FCS + deletion | -0.02 | 0.18 | 0.18 | 0.951 | 0.03 | 0.07 | 0.24 | 0.24 | 0.942 | 0.06 |
|  |  |  |  |  | CCA | 0.02 | 0.23 | 0.22 | 0.956 | 0.05 | 0.01 | 0.23 | 0.23 | 0.949 | 0.05 |

Abbreviations: MVNI = multivariate normal imputation, FCS = fully conditional specification, CCA = complete case analysis, Avg SE = average standard error, Emp SE = empirical standard error, MSE = mean square error.

^a^ $\lambda$ indicates the strength of the missing data mechanism, 1 = moderate, 2 = strong.

**Web Table 2** Full Simulation Results for $X_{1}$ and $X_{2}$ continuous

| Missing data mechanism | $\lambda$^a^ | Outcome prevalence | $\beta_{1},\beta_{2}$ | $\mathrm{Corr}\left( X_{1},X_{2} \right)$ | Method | $\beta_{1}$ | | | | | $\beta_{2}$ | | | | |
| --- | --- | --- | --- | --- | --- | --- | --- | --- | --- | --- | --- | --- | --- | --- | --- |
|  |  |  |  |  |  | Bias | Avg SE | Emp SE | Coverage | MSE | Bias | Avg SE | Emp SE | Coverage | MSE |
| Coordinated | 1 | 0.10 | Log(2) | 0.30 | MVNI | -0.18 | 0.29 | 0.24 | 0.952 | 0.09 | -0.11 | 0.33 | 0.29 | 0.963 | 0.10 |
|  |  |  |  |  | MVNI + deletion | -0.01 | 0.33 | 0.31 | 0.962 | 0.10 | -0.01 | 0.35 | 0.34 | 0.958 | 0.12 |
|  |  |  |  |  | FCS | -0.02 | 0.31 | 0.30 | 0.958 | 0.09 | -0.02 | 0.34 | 0.33 | 0.955 | 0.11 |
|  |  |  |  |  | FCS + deletion | -0.00 | 0.33 | 0.31 | 0.964 | 0.10 | -0.01 | 0.35 | 0.34 | 0.961 | 0.11 |
|  |  |  |  |  | CCA | -0.00 | 0.39 | 0.39 | 0.935 | 0.15 | 0.01 | 0.34 | 0.34 | 0.940 | 0.12 |
| Coordinated | 1 | 0.10 | Log(2) | 0.70 | MVNI | -0.20 | 0.42 | 0.36 | 0.953 | 0.17 | -0.11 | 0.45 | 0.40 | 0.971 | 0.17 |
|  |  |  |  |  | MVNI + deletion | 0.01 | 0.47 | 0.45 | 0.963 | 0.20 | -0.00 | 0.49 | 0.47 | 0.958 | 0.22 |
|  |  |  |  |  | FCS | -0.01 | 0.44 | 0.43 | 0.953 | 0.19 | -0.03 | 0.47 | 0.46 | 0.955 | 0.21 |
|  |  |  |  |  | FCS + deletion | 0.01 | 0.47 | 0.45 | 0.959 | 0.20 | -0.01 | 0.49 | 0.47 | 0.963 | 0.22 |
|  |  |  |  |  | CCA | 0.00 | 0.51 | 0.52 | 0.935 | 0.27 | 0.01 | 0.47 | 0.48 | 0.943 | 0.23 |
| Coordinated | 1 | 0.10 | Log(3) | 0.30 | MVNI | -0.29 | 0.30 | 0.24 | 0.892 | 0.15 | -0.21 | 0.34 | 0.29 | 0.943 | 0.13 |
|  |  |  |  |  | MVNI + deletion | -0.01 | 0.35 | 0.32 | 0.969 | 0.10 | -0.04 | 0.37 | 0.35 | 0.964 | 0.12 |
|  |  |  |  |  | FCS | -0.05 | 0.32 | 0.30 | 0.963 | 0.09 | -0.09 | 0.35 | 0.33 | 0.953 | 0.12 |
|  |  |  |  |  | FCS + deletion | -0.01 | 0.35 | 0.32 | 0.972 | 0.10 | -0.04 | 0.37 | 0.35 | 0.958 | 0.12 |
|  |  |  |  |  | CCA | 0.01 | 0.41 | 0.42 | 0.938 | 0.18 | -0.01 | 0.35 | 0.36 | 0.940 | 0.13 |
| Coordinated | 1 | 0.10 | Log(3) | 0.70 | MVNI | -0.31 | 0.45 | 0.37 | 0.939 | 0.23 | -0.23 | 0.47 | 0.40 | 0.963 | 0.22 |
|  |  |  |  |  | MVNI + deletion | 0.03 | 0.50 | 0.47 | 0.970 | 0.22 | -0.06 | 0.52 | 0.48 | 0.970 | 0.23 |
|  |  |  |  |  | FCS | -0.02 | 0.46 | 0.44 | 0.965 | 0.19 | -0.12 | 0.48 | 0.45 | 0.965 | 0.22 |
|  |  |  |  |  | FCS + deletion | 0.03 | 0.50 | 0.47 | 0.971 | 0.22 | -0.06 | 0.52 | 0.48 | 0.969 | 0.23 |
|  |  |  |  |  | CCA | 0.02 | 0.55 | 0.56 | 0.940 | 0.32 | 0.00 | 0.50 | 0.51 | 0.942 | 0.26 |
| Coordinated | 1 | 0.30 | Log(2) | 0.30 | MVNI | -0.11 | 0.16 | 0.13 | 0.956 | 0.03 | -0.10 | 0.18 | 0.14 | 0.956 | 0.03 |
|  |  |  |  |  | MVNI + deletion | -0.00 | 0.19 | 0.15 | 0.987 | 0.02 | -0.02 | 0.19 | 0.16 | 0.981 | 0.03 |
|  |  |  |  |  | FCS | -0.04 | 0.17 | 0.14 | 0.975 | 0.02 | -0.06 | 0.18 | 0.15 | 0.965 | 0.03 |
|  |  |  |  |  | FCS + deletion | -0.00 | 0.19 | 0.15 | 0.985 | 0.02 | -0.02 | 0.19 | 0.16 | 0.983 | 0.03 |
|  |  |  |  |  | CCA | 0.00 | 0.20 | 0.19 | 0.953 | 0.04 | 0.01 | 0.17 | 0.17 | 0.950 | 0.03 |
| Coordinated | 1 | 0.30 | Log(2) | 0.70 | MVNI | -0.10 | 0.23 | 0.19 | 0.968 | 0.05 | -0.12 | 0.24 | 0.20 | 0.959 | 0.05 |
|  |  |  |  |  | MVNI + deletion | 0.02 | 0.26 | 0.22 | 0.984 | 0.05 | -0.04 | 0.26 | 0.22 | 0.986 | 0.05 |
|  |  |  |  |  | FCS | -0.02 | 0.23 | 0.21 | 0.976 | 0.04 | -0.08 | 0.24 | 0.21 | 0.971 | 0.05 |
|  |  |  |  |  | FCS + deletion | 0.02 | 0.26 | 0.22 | 0.983 | 0.05 | -0.04 | 0.26 | 0.22 | 0.986 | 0.05 |
|  |  |  |  |  | CCA | 0.00 | 0.26 | 0.26 | 0.942 | 0.07 | 0.01 | 0.24 | 0.24 | 0.953 | 0.06 |
| Coordinated | 1 | 0.30 | Log(3) | 0.30 | MVNI | -0.22 | 0.17 | 0.13 | 0.820 | 0.07 | -0.22 | 0.18 | 0.14 | 0.842 | 0.07 |
|  |  |  |  |  | MVNI + deletion | -0.06 | 0.20 | 0.16 | 0.983 | 0.03 | -0.09 | 0.20 | 0.16 | 0.970 | 0.03 |
|  |  |  |  |  | FCS | -0.12 | 0.17 | 0.14 | 0.939 | 0.04 | -0.16 | 0.18 | 0.15 | 0.906 | 0.05 |
|  |  |  |  |  | FCS + deletion | -0.06 | 0.20 | 0.16 | 0.981 | 0.03 | -0.10 | 0.20 | 0.16 | 0.968 | 0.03 |
|  |  |  |  |  | CCA | 0.00 | 0.20 | 0.21 | 0.947 | 0.04 | -0.00 | 0.18 | 0.18 | 0.956 | 0.03 |
| Coordinated | 1 | 0.30 | Log(3) | 0.70 | MVNI | -0.17 | 0.24 | 0.19 | 0.946 | 0.07 | -0.25 | 0.24 | 0.19 | 0.887 | 0.10 |
|  |  |  |  |  | MVNI + deletion | -0.00 | 0.28 | 0.22 | 0.985 | 0.05 | -0.12 | 0.27 | 0.22 | 0.969 | 0.06 |
|  |  |  |  |  | FCS | -0.05 | 0.24 | 0.21 | 0.977 | 0.05 | -0.19 | 0.24 | 0.21 | 0.921 | 0.08 |
|  |  |  |  |  | FCS + deletion | 0.01 | 0.28 | 0.22 | 0.987 | 0.05 | -0.12 | 0.27 | 0.22 | 0.966 | 0.06 |
|  |  |  |  |  | CCA | 0.01 | 0.26 | 0.27 | 0.947 | 0.07 | 0.01 | 0.24 | 0.24 | 0.947 | 0.06 |
| Coordinated | 2 | 0.10 | Log(2) | 0.30 | MVNI | -0.27 | 0.31 | 0.24 | 0.933 | 0.13 | -0.13 | 0.33 | 0.28 | 0.965 | 0.09 |
|  |  |  |  |  | MVNI + deletion | -0.01 | 0.38 | 0.36 | 0.969 | 0.13 | -0.02 | 0.35 | 0.33 | 0.967 | 0.11 |
|  |  |  |  |  | FCS | -0.03 | 0.35 | 0.33 | 0.963 | 0.11 | -0.04 | 0.34 | 0.32 | 0.964 | 0.10 |
|  |  |  |  |  | FCS + deletion | -0.01 | 0.38 | 0.36 | 0.969 | 0.13 | -0.02 | 0.35 | 0.33 | 0.966 | 0.11 |
|  |  |  |  |  | CCA | -0.01 | 0.45 | 0.45 | 0.945 | 0.20 | -0.01 | 0.34 | 0.33 | 0.947 | 0.11 |
| Coordinated | 2 | 0.10 | Log(2) | 0.70 | MVNI | -0.32 | 0.44 | 0.35 | 0.939 | 0.23 | -0.13 | 0.45 | 0.40 | 0.969 | 0.17 |
|  |  |  |  |  | MVNI + deletion | 0.01 | 0.51 | 0.47 | 0.964 | 0.23 | -0.01 | 0.49 | 0.47 | 0.959 | 0.22 |
|  |  |  |  |  | FCS | -0.02 | 0.47 | 0.45 | 0.964 | 0.20 | -0.05 | 0.46 | 0.45 | 0.961 | 0.20 |
|  |  |  |  |  | FCS + deletion | 0.01 | 0.51 | 0.47 | 0.963 | 0.23 | -0.01 | 0.49 | 0.47 | 0.962 | 0.22 |
|  |  |  |  |  | CCA | 0.02 | 0.57 | 0.58 | 0.941 | 0.34 | -0.00 | 0.47 | 0.48 | 0.940 | 0.23 |
| Coordinated | 2 | 0.10 | Log(3) | 0.30 | MVNI | -0.46 | 0.32 | 0.25 | 0.758 | 0.27 | -0.21 | 0.35 | 0.31 | 0.934 | 0.14 |
|  |  |  |  |  | MVNI + deletion | 0.00 | 0.42 | 0.39 | 0.964 | 0.15 | -0.03 | 0.38 | 0.37 | 0.959 | 0.14 |
|  |  |  |  |  | FCS | -0.07 | 0.35 | 0.34 | 0.953 | 0.12 | -0.11 | 0.35 | 0.34 | 0.952 | 0.13 |
|  |  |  |  |  | FCS + deletion | 0.01 | 0.42 | 0.39 | 0.969 | 0.15 | -0.03 | 0.38 | 0.37 | 0.958 | 0.13 |
|  |  |  |  |  | CCA | 0.03 | 0.50 | 0.51 | 0.944 | 0.26 | -0.00 | 0.36 | 0.38 | 0.935 | 0.15 |
| Coordinated | 2 | 0.10 | Log(3) | 0.70 | MVNI | -0.56 | 0.48 | 0.39 | 0.838 | 0.47 | -0.22 | 0.50 | 0.43 | 0.958 | 0.24 |
|  |  |  |  |  | MVNI + deletion | 0.01 | 0.58 | 0.55 | 0.965 | 0.30 | -0.03 | 0.55 | 0.53 | 0.959 | 0.28 |
|  |  |  |  |  | FCS | -0.08 | 0.51 | 0.49 | 0.961 | 0.25 | -0.14 | 0.50 | 0.48 | 0.950 | 0.25 |
|  |  |  |  |  | FCS + deletion | 0.02 | 0.58 | 0.55 | 0.964 | 0.30 | -0.04 | 0.55 | 0.53 | 0.961 | 0.28 |
|  |  |  |  |  | CCA | 0.01 | 0.66 | 0.67 | 0.943 | 0.45 | 0.01 | 0.53 | 0.55 | 0.936 | 0.30 |
| Coordinated | 2 | 0.30 | Log(2) | 0.30 | MVNI | -0.16 | 0.17 | 0.14 | 0.919 | 0.04 | -0.12 | 0.17 | 0.14 | 0.940 | 0.03 |
|  |  |  |  |  | MVNI + deletion | 0.00 | 0.22 | 0.18 | 0.984 | 0.03 | -0.02 | 0.19 | 0.17 | 0.975 | 0.03 |
|  |  |  |  |  | FCS | -0.06 | 0.18 | 0.16 | 0.970 | 0.03 | -0.08 | 0.18 | 0.15 | 0.957 | 0.03 |
|  |  |  |  |  | FCS + deletion | 0.00 | 0.22 | 0.18 | 0.985 | 0.03 | -0.02 | 0.19 | 0.17 | 0.978 | 0.03 |
|  |  |  |  |  | CCA | 0.00 | 0.23 | 0.23 | 0.947 | 0.05 | 0.00 | 0.17 | 0.17 | 0.949 | 0.03 |
| Coordinated | 2 | 0.30 | Log(2) | 0.70 | MVNI | -0.17 | 0.24 | 0.20 | 0.934 | 0.07 | -0.14 | 0.24 | 0.20 | 0.945 | 0.06 |
|  |  |  |  |  | MVNI + deletion | 0.02 | 0.29 | 0.25 | 0.977 | 0.06 | -0.04 | 0.27 | 0.24 | 0.969 | 0.06 |
|  |  |  |  |  | FCS | -0.05 | 0.24 | 0.22 | 0.966 | 0.05 | -0.10 | 0.24 | 0.22 | 0.951 | 0.06 |
|  |  |  |  |  | FCS + deletion | 0.02 | 0.29 | 0.25 | 0.978 | 0.06 | -0.04 | 0.27 | 0.24 | 0.968 | 0.06 |
|  |  |  |  |  | CCA | -0.00 | 0.30 | 0.31 | 0.941 | 0.09 | 0.00 | 0.24 | 0.25 | 0.940 | 0.06 |
| Coordinated | 2 | 0.30 | Log(3) | 0.30 | MVNI | -0.29 | 0.18 | 0.14 | 0.689 | 0.11 | -0.24 | 0.18 | 0.15 | 0.789 | 0.08 |
|  |  |  |  |  | MVNI + deletion | -0.04 | 0.24 | 0.19 | 0.984 | 0.04 | -0.08 | 0.20 | 0.17 | 0.970 | 0.03 |
|  |  |  |  |  | FCS | -0.16 | 0.18 | 0.15 | 0.909 | 0.05 | -0.19 | 0.18 | 0.15 | 0.849 | 0.06 |
|  |  |  |  |  | FCS + deletion | -0.04 | 0.24 | 0.19 | 0.983 | 0.04 | -0.08 | 0.20 | 0.17 | 0.969 | 0.03 |
|  |  |  |  |  | CCA | 0.01 | 0.25 | 0.25 | 0.951 | 0.06 | 0.01 | 0.18 | 0.18 | 0.948 | 0.03 |
| Coordinated | 2 | 0.30 | Log(3) | 0.70 | MVNI | -0.26 | 0.26 | 0.20 | 0.890 | 0.11 | -0.27 | 0.25 | 0.20 | 0.859 | 0.11 |
|  |  |  |  |  | MVNI + deletion | 0.01 | 0.31 | 0.26 | 0.978 | 0.07 | -0.11 | 0.28 | 0.23 | 0.963 | 0.07 |
|  |  |  |  |  | FCS | -0.09 | 0.26 | 0.22 | 0.962 | 0.06 | -0.24 | 0.24 | 0.21 | 0.878 | 0.10 |
|  |  |  |  |  | FCS + deletion | 0.02 | 0.31 | 0.26 | 0.980 | 0.07 | -0.12 | 0.28 | 0.23 | 0.963 | 0.07 |
|  |  |  |  |  | CCA | 0.02 | 0.32 | 0.32 | 0.951 | 0.11 | -0.00 | 0.25 | 0.26 | 0.950 | 0.07 |
| Opposite | 1 | 0.10 | Log(2) | 0.30 | MVNI | -0.18 | 0.29 | 0.24 | 0.948 | 0.09 | -0.09 | 0.32 | 0.29 | 0.962 | 0.09 |
|  |  |  |  |  | MVNI + deletion | -0.01 | 0.33 | 0.31 | 0.961 | 0.10 | 0.01 | 0.35 | 0.34 | 0.960 | 0.11 |
|  |  |  |  |  | FCS | -0.02 | 0.31 | 0.30 | 0.956 | 0.09 | -0.01 | 0.34 | 0.33 | 0.962 | 0.11 |
|  |  |  |  |  | FCS + deletion | -0.01 | 0.33 | 0.31 | 0.961 | 0.10 | 0.01 | 0.35 | 0.34 | 0.962 | 0.11 |
|  |  |  |  |  | CCA | 0.00 | 0.37 | 0.39 | 0.936 | 0.15 | 0.01 | 0.33 | 0.33 | 0.944 | 0.11 |
| Opposite | 1 | 0.10 | Log(2) | 0.70 | MVNI | -0.20 | 0.41 | 0.35 | 0.961 | 0.16 | -0.10 | 0.43 | 0.39 | 0.969 | 0.16 |
|  |  |  |  |  | MVNI + deletion | 0.00 | 0.46 | 0.43 | 0.966 | 0.18 | 0.00 | 0.47 | 0.45 | 0.961 | 0.21 |
|  |  |  |  |  | FCS | -0.01 | 0.43 | 0.41 | 0.964 | 0.17 | -0.02 | 0.45 | 0.44 | 0.961 | 0.20 |
|  |  |  |  |  | FCS + deletion | 0.01 | 0.46 | 0.43 | 0.965 | 0.18 | -0.00 | 0.47 | 0.45 | 0.960 | 0.21 |
|  |  |  |  |  | CCA | 0.00 | 0.47 | 0.48 | 0.946 | 0.23 | -0.00 | 0.43 | 0.45 | 0.943 | 0.20 |
| Opposite | 1 | 0.10 | Log(3) | 0.30 | MVNI | -0.29 | 0.30 | 0.23 | 0.896 | 0.14 | -0.15 | 0.33 | 0.28 | 0.958 | 0.10 |
|  |  |  |  |  | MVNI + deletion | -0.01 | 0.34 | 0.31 | 0.970 | 0.10 | 0.02 | 0.36 | 0.33 | 0.966 | 0.11 |
|  |  |  |  |  | FCS | -0.04 | 0.31 | 0.29 | 0.963 | 0.09 | -0.03 | 0.34 | 0.31 | 0.965 | 0.10 |
|  |  |  |  |  | FCS + deletion | -0.00 | 0.34 | 0.31 | 0.973 | 0.10 | 0.01 | 0.36 | 0.32 | 0.966 | 0.11 |
|  |  |  |  |  | CCA | -0.00 | 0.37 | 0.38 | 0.947 | 0.14 | 0.01 | 0.32 | 0.32 | 0.949 | 0.10 |
| Opposite | 1 | 0.10 | Log(3) | 0.70 | MVNI | -0.34 | 0.42 | 0.35 | 0.922 | 0.24 | -0.16 | 0.45 | 0.38 | 0.972 | 0.17 |
|  |  |  |  |  | MVNI + deletion | -0.01 | 0.48 | 0.44 | 0.972 | 0.19 | 0.02 | 0.49 | 0.45 | 0.969 | 0.21 |
|  |  |  |  |  | FCS | -0.05 | 0.44 | 0.41 | 0.961 | 0.17 | -0.03 | 0.46 | 0.43 | 0.964 | 0.19 |
|  |  |  |  |  | FCS + deletion | -0.01 | 0.48 | 0.44 | 0.968 | 0.19 | 0.02 | 0.49 | 0.45 | 0.970 | 0.21 |
|  |  |  |  |  | CCA | -0.01 | 0.48 | 0.48 | 0.941 | 0.23 | 0.01 | 0.44 | 0.45 | 0.946 | 0.20 |
| Opposite | 1 | 0.30 | Log(2) | 0.30 | MVNI | -0.10 | 0.16 | 0.13 | 0.952 | 0.03 | -0.06 | 0.18 | 0.15 | 0.975 | 0.03 |
|  |  |  |  |  | MVNI + deletion | -0.00 | 0.19 | 0.16 | 0.983 | 0.02 | 0.02 | 0.20 | 0.17 | 0.976 | 0.03 |
|  |  |  |  |  | FCS | -0.03 | 0.16 | 0.15 | 0.971 | 0.02 | -0.01 | 0.18 | 0.16 | 0.970 | 0.03 |
|  |  |  |  |  | FCS + deletion | 0.00 | 0.19 | 0.16 | 0.984 | 0.02 | 0.02 | 0.20 | 0.17 | 0.976 | 0.03 |
|  |  |  |  |  | CCA | 0.00 | 0.19 | 0.19 | 0.941 | 0.04 | 0.00 | 0.16 | 0.17 | 0.939 | 0.03 |
| Opposite | 1 | 0.30 | Log(2) | 0.70 | MVNI | -0.12 | 0.23 | 0.19 | 0.960 | 0.05 | -0.06 | 0.24 | 0.20 | 0.977 | 0.05 |
|  |  |  |  |  | MVNI + deletion | -0.01 | 0.26 | 0.22 | 0.979 | 0.05 | 0.02 | 0.27 | 0.23 | 0.974 | 0.05 |
|  |  |  |  |  | FCS | -0.04 | 0.23 | 0.20 | 0.970 | 0.04 | -0.01 | 0.24 | 0.22 | 0.972 | 0.05 |
|  |  |  |  |  | FCS + deletion | -0.01 | 0.26 | 0.22 | 0.981 | 0.05 | 0.02 | 0.27 | 0.23 | 0.976 | 0.05 |
|  |  |  |  |  | CCA | 0.01 | 0.24 | 0.24 | 0.948 | 0.06 | -0.00 | 0.22 | 0.22 | 0.945 | 0.05 |
| Opposite | 1 | 0.30 | Log(3) | 0.30 | MVNI | -0.14 | 0.17 | 0.13 | 0.940 | 0.04 | -0.10 | 0.19 | 0.15 | 0.958 | 0.03 |
|  |  |  |  |  | MVNI + deletion | 0.02 | 0.20 | 0.16 | 0.989 | 0.03 | 0.02 | 0.21 | 0.17 | 0.981 | 0.03 |
|  |  |  |  |  | FCS | -0.02 | 0.17 | 0.14 | 0.987 | 0.02 | -0.03 | 0.19 | 0.16 | 0.979 | 0.03 |
|  |  |  |  |  | FCS + deletion | 0.02 | 0.20 | 0.16 | 0.989 | 0.03 | 0.01 | 0.21 | 0.17 | 0.986 | 0.03 |
|  |  |  |  |  | CCA | 0.01 | 0.19 | 0.19 | 0.943 | 0.04 | 0.01 | 0.17 | 0.17 | 0.951 | 0.03 |
| Opposite | 1 | 0.30 | Log(3) | 0.70 | MVNI | -0.17 | 0.23 | 0.18 | 0.946 | 0.06 | -0.11 | 0.25 | 0.20 | 0.956 | 0.05 |
|  |  |  |  |  | MVNI + deletion | 0.01 | 0.26 | 0.21 | 0.983 | 0.04 | 0.02 | 0.27 | 0.23 | 0.979 | 0.05 |
|  |  |  |  |  | FCS | -0.04 | 0.23 | 0.19 | 0.981 | 0.04 | -0.04 | 0.25 | 0.22 | 0.970 | 0.05 |
|  |  |  |  |  | FCS + deletion | 0.01 | 0.26 | 0.21 | 0.985 | 0.04 | 0.01 | 0.27 | 0.23 | 0.979 | 0.05 |
|  |  |  |  |  | CCA | 0.00 | 0.23 | 0.24 | 0.940 | 0.06 | 0.00 | 0.22 | 0.23 | 0.933 | 0.05 |
| Opposite | 2 | 0.10 | Log(2) | 0.30 | MVNI | -0.27 | 0.31 | 0.24 | 0.932 | 0.13 | -0.09 | 0.34 | 0.32 | 0.961 | 0.11 |
|  |  |  |  |  | MVNI + deletion | -0.00 | 0.38 | 0.36 | 0.966 | 0.13 | 0.02 | 0.37 | 0.37 | 0.948 | 0.14 |
|  |  |  |  |  | FCS | -0.02 | 0.35 | 0.33 | 0.959 | 0.11 | -0.01 | 0.35 | 0.36 | 0.946 | 0.13 |
|  |  |  |  |  | FCS + deletion | 0.00 | 0.38 | 0.36 | 0.965 | 0.13 | 0.02 | 0.37 | 0.37 | 0.946 | 0.14 |
|  |  |  |  |  | CCA | 0.01 | 0.49 | 0.49 | 0.939 | 0.24 | 0.01 | 0.34 | 0.37 | 0.931 | 0.14 |
| Opposite | 2 | 0.10 | Log(2) | 0.70 | MVNI | -0.31 | 0.44 | 0.36 | 0.939 | 0.23 | -0.12 | 0.46 | 0.41 | 0.963 | 0.18 |
|  |  |  |  |  | MVNI + deletion | 0.03 | 0.52 | 0.49 | 0.962 | 0.24 | -0.01 | 0.50 | 0.48 | 0.956 | 0.24 |
|  |  |  |  |  | FCS | -0.00 | 0.47 | 0.45 | 0.960 | 0.21 | -0.05 | 0.48 | 0.46 | 0.957 | 0.21 |
|  |  |  |  |  | FCS + deletion | 0.04 | 0.52 | 0.49 | 0.965 | 0.24 | -0.01 | 0.50 | 0.48 | 0.962 | 0.23 |
|  |  |  |  |  | CCA | 0.03 | 0.59 | 0.59 | 0.942 | 0.35 | -0.02 | 0.46 | 0.48 | 0.939 | 0.23 |
| Opposite | 2 | 0.10 | Log(3) | 0.30 | MVNI | -0.45 | 0.32 | 0.25 | 0.783 | 0.27 | -0.18 | 0.35 | 0.30 | 0.952 | 0.12 |
|  |  |  |  |  | MVNI + deletion | 0.01 | 0.41 | 0.39 | 0.967 | 0.15 | 0.01 | 0.38 | 0.36 | 0.959 | 0.13 |
|  |  |  |  |  | FCS | -0.05 | 0.35 | 0.34 | 0.953 | 0.12 | -0.06 | 0.36 | 0.34 | 0.951 | 0.12 |
|  |  |  |  |  | FCS + deletion | 0.02 | 0.41 | 0.39 | 0.966 | 0.15 | 0.01 | 0.38 | 0.36 | 0.962 | 0.13 |
|  |  |  |  |  | CCA | 0.01 | 0.50 | 0.51 | 0.946 | 0.26 | -0.00 | 0.35 | 0.35 | 0.938 | 0.13 |
| Opposite | 2 | 0.10 | Log(3) | 0.70 | MVNI | -0.58 | 0.47 | 0.37 | 0.830 | 0.47 | -0.17 | 0.48 | 0.42 | 0.961 | 0.21 |
|  |  |  |  |  | MVNI + deletion | -0.00 | 0.56 | 0.52 | 0.966 | 0.28 | 0.02 | 0.53 | 0.51 | 0.959 | 0.26 |
|  |  |  |  |  | FCS | -0.08 | 0.48 | 0.46 | 0.961 | 0.22 | -0.07 | 0.49 | 0.47 | 0.959 | 0.22 |
|  |  |  |  |  | FCS + deletion | 0.01 | 0.56 | 0.52 | 0.971 | 0.27 | 0.01 | 0.53 | 0.51 | 0.962 | 0.26 |
|  |  |  |  |  | CCA | 0.00 | 0.60 | 0.62 | 0.939 | 0.39 | 0.01 | 0.48 | 0.50 | 0.938 | 0.25 |
| Opposite | 2 | 0.30 | Log(2) | 0.30 | MVNI | -0.15 | 0.17 | 0.14 | 0.924 | 0.04 | -0.07 | 0.19 | 0.16 | 0.963 | 0.03 |
|  |  |  |  |  | MVNI + deletion | 0.01 | 0.22 | 0.18 | 0.981 | 0.03 | 0.02 | 0.21 | 0.18 | 0.972 | 0.03 |
|  |  |  |  |  | FCS | -0.05 | 0.18 | 0.16 | 0.969 | 0.03 | -0.03 | 0.19 | 0.17 | 0.968 | 0.03 |
|  |  |  |  |  | FCS + deletion | 0.01 | 0.22 | 0.19 | 0.980 | 0.03 | 0.02 | 0.21 | 0.18 | 0.971 | 0.03 |
|  |  |  |  |  | CCA | 0.01 | 0.25 | 0.24 | 0.954 | 0.06 | -0.01 | 0.17 | 0.18 | 0.942 | 0.03 |
| Opposite | 2 | 0.30 | Log(2) | 0.70 | MVNI | -0.19 | 0.24 | 0.19 | 0.933 | 0.07 | -0.07 | 0.25 | 0.21 | 0.972 | 0.05 |
|  |  |  |  |  | MVNI + deletion | -0.01 | 0.29 | 0.24 | 0.981 | 0.06 | 0.03 | 0.28 | 0.24 | 0.981 | 0.06 |
|  |  |  |  |  | FCS | -0.08 | 0.24 | 0.21 | 0.966 | 0.05 | -0.03 | 0.25 | 0.22 | 0.970 | 0.05 |
|  |  |  |  |  | FCS + deletion | -0.01 | 0.29 | 0.24 | 0.982 | 0.06 | 0.03 | 0.28 | 0.24 | 0.978 | 0.06 |
|  |  |  |  |  | CCA | 0.00 | 0.29 | 0.29 | 0.945 | 0.09 | -0.00 | 0.23 | 0.23 | 0.954 | 0.05 |
| Opposite | 2 | 0.30 | Log(3) | 0.30 | MVNI | -0.18 | 0.18 | 0.13 | 0.912 | 0.05 | -0.09 | 0.20 | 0.16 | 0.962 | 0.03 |
|  |  |  |  |  | MVNI + deletion | 0.04 | 0.23 | 0.18 | 0.983 | 0.04 | 0.05 | 0.22 | 0.18 | 0.977 | 0.04 |
|  |  |  |  |  | FCS | -0.03 | 0.18 | 0.15 | 0.975 | 0.02 | -0.03 | 0.20 | 0.17 | 0.972 | 0.03 |
|  |  |  |  |  | FCS + deletion | 0.05 | 0.23 | 0.18 | 0.982 | 0.04 | 0.04 | 0.22 | 0.18 | 0.978 | 0.04 |
|  |  |  |  |  | CCA | 0.01 | 0.24 | 0.25 | 0.946 | 0.06 | 0.01 | 0.17 | 0.18 | 0.945 | 0.03 |
| Opposite | 2 | 0.30 | Log(3) | 0.70 | MVNI | -0.25 | 0.24 | 0.19 | 0.886 | 0.10 | -0.07 | 0.26 | 0.20 | 0.981 | 0.05 |
|  |  |  |  |  | MVNI + deletion | 0.01 | 0.30 | 0.25 | 0.983 | 0.06 | 0.06 | 0.29 | 0.23 | 0.980 | 0.06 |
|  |  |  |  |  | FCS | -0.07 | 0.24 | 0.21 | 0.974 | 0.05 | -0.02 | 0.26 | 0.22 | 0.980 | 0.05 |
|  |  |  |  |  | FCS + deletion | 0.02 | 0.29 | 0.25 | 0.983 | 0.06 | 0.05 | 0.29 | 0.23 | 0.982 | 0.06 |
|  |  |  |  |  | CCA | -0.00 | 0.29 | 0.29 | 0.945 | 0.08 | 0.01 | 0.23 | 0.22 | 0.949 | 0.05 |

Abbreviations: MVNI = multivariate normal imputation, FCS = fully conditional specification, CCA = complete case analysis, Avg SE = average standard error, Emp SE = empirical standard error, MSE = mean square error.^a^

$\lambda$ indicates the strength of the missing data mechanism, 1 = moderate, 2 = strong.

**Web Table 3** Bias in “Null-Case” Scenarios with $X_{1}$ and $X_{2}$ Binary, Coordinated Missing Data Mechanism

| Simulation scenario | Parameter | MVNI | MVNI + deletion | FCS | FCS + deletion |
| --- | --- | --- | --- | --- | --- |
| 1. Outcome prevalence = 0.10, RR$\left( X_{1},X_{2} \right)=3$, $\beta_{1}=\beta_{2}=log(3)$, $\lambda=2$ | $\beta_{1}$ | -0.03 | 0.10 | 0.05 | 0.04 |
|  | $\beta_{2}$ | -0.28 | -0.10 | 0.00 | 0.02 |
| 2. As in (1), but with $\mathrm{RR}\left( X_{1},X_{2} \right)=1$ | $\beta_{1}$ | -0.14 | 0.01 | 0.01 | 0.01 |
|  | $\beta_{2}$ | -0.28 | -0.08 | 0.01 | 0.03 |
| 3. As in (1), but with $\beta_{1}=0$ | $\beta_{1}$ | 0.02 | 0.05 | 0.01 | 0.01 |
|  | $\beta_{2}$ | -0.17 | -0.05 | 0.00 | 0.01 |
| 4. As in (1), but with $\beta_{2}=0$ | $\beta_{1}$ | -0.15 | 0.00 | 0.00 | 0.00 |
|  | $\beta_{2}$ | 0.02 | 0.02 | 0.01 | 0.01 |
| 5. As in (1), but with $\beta_{1}=\beta_{2}=0$ | $\beta_{1}$ | 0.00 | 0.00 | 0.00 | 0.00 |
|  | $\beta_{2}$ | 0.00 | -0.01 | -0.02 | -0.01 |
| 6. As in (1), but with $\lambda=0$ (MCAR) | $\beta_{1}$ | -0.12 | 0.11 | 0.04 | 0.04 |
|  | $\beta_{2}$ | -0.28 | -0.10 | 0.05 | 0.06 |
| 7. Outcome prevalence = 0.30, RR$\left( X_{1},X_{2} \right)=3$, $\beta_{1}=\beta_{2}=log(3)$, $\lambda=2$ | $\beta_{1}$ | 0.07 | 0.07 | 0.04 | 0.02 |
|  | $\beta_{2}$ | -0.31 | -0.14 | -0.10 | -0.04 |
| 8. As in (7), but with $\mathrm{RR}\left( X_{1},X_{2} \right)=1$ | $\beta_{1}$ | -0.07 | -0.01 | -0.01 | -0.01 |
|  | $\beta_{2}$ | -0.32 | -0.16 | -0.12 | -0.07 |
| 9. As in (7), but with $\beta_{1}=0$ | $\beta_{1}$ | 0.03 | 0.03 | 0.00 | 0.00 |
|  | $\beta_{2}$ | -0.12 | -0.05 | 0.01 | 0.01 |
| 10. As in (7), but with $\beta_{2}=0$ | $\beta_{1}$ | -0.06 | 0.00 | 0.00 | 0.00 |
|  | $\beta_{2}$ | 0.00 | 0.00 | 0.00 | 0.00 |
| 11. As in (7), but with $\beta_{1}=\beta_{2}=0$ | $\beta_{1}$ | 0.00 | 0.00 | 0.00 | 0.00 |
|  | $\beta_{2}$ | 0.00 | 0.00 | 0.00 | 0.00 |
| 12. As in (7), but with $\lambda=0$ (MCAR) | $\beta_{1}$ | -0.04 | 0.07 | 0.00 | 0.01 |
|  | $\beta_{2}$ | -0.21 | -0.11 | 0.00 | 0.01 |

Abbreviations: RR = relative risk, MCAR = missing completely at random, MVNI = multivariate normal imputation, FCS = fully conditional specification.

**Web Table 4** Bias in “Null-Case” Scenarios with $X_{1}$ and $X_{2}$ Continuous, Coordinated Missing Data Mechanism

| Simulation scenario | Parameter | MVNI | MVNI + deletion | FCS | FCS + deletion |
| --- | --- | --- | --- | --- | --- |
| 1. Outcome prevalence = 0.10, $C\mathrm{orr}\left( X_{1},X_{2} \right)=0.70$, $\beta_{1}=\beta_{2}=log(3)$, $\lambda=2$ | $\beta_{1}$ | -0.56 | 0.01 | -0.08 | 0.02 |
|  | $\beta_{2}$ | -0.22 | -0.03 | -0.14 | -0.04 |
| 2. As in (1.), but with $C\mathrm{orr}\left( X_{1},X_{2} \right)=0$ | $\beta_{1}$ | -0.38 | 0.00 | -0.06 | 0.00 |
|  | $\beta_{2}$ | -0.20 | -0.02 | -0.08 | -0.02 |
| 3. As in (1.), but with $\beta_{1}=0$ | $\beta_{1}$ | -0.13 | 0.01 | 0.01 | 0.01 |
|  | $\beta_{2}$ | -0.17 | 0.00 | -0.04 | 0.00 |
| 4. As in (1.), but with $\beta_{2}=0$ | $\beta_{1}$ | -0.37 | 0.01 | -0.04 | 0.01 |
|  | $\beta_{2}$ | 0.00 | 0.00 | 0.00 | -0.01 |
| 5. As in (1.), but with $\beta_{1}=\beta_{2}=0$ | $\beta_{1}$ | 0.00 | 0.00 | 0.01 | 0.00 |
|  | $\beta_{2}$ | 0.00 | 0.00 | 0.00 | 0.00 |
| 6. As in (1.), but with $\lambda=0$ (MCAR) | $\beta_{1}$ | -0.15 | 0.00 | 0.00 | 0.00 |
|  | $\beta_{2}$ | -0.17 | -0.03 | -0.03 | -0.03 |
| 7. Outcome prevalence = 0.30, $Co\mathrm{rr}\left( X_{1},X_{2} \right)=0.70$, $\beta_{1}=\beta_{2}=log(3)$, $\lambda=2$ | $\beta_{1}$ | -0.26 | 0.01 | -0.09 | 0.02 |
|  | $\beta_{2}$ | -0.27 | -0.11 | -0.24 | -0.12 |
| 8. As in (7.), but with $C\mathrm{orr}\left( X_{1},X_{2} \right)=0$ | $\beta_{1}$ | -0.27 | -0.05 | -0.15 | -0.05 |
|  | $\beta_{2}$ | -0.21 | -0.06 | -0.16 | -0.06 |
| 9. As in (7.), but with $\beta_{1}=0$ | $\beta_{1}$ | -0.04 | 0.02 | 0.01 | 0.02 |
|  | $\beta_{2}$ | -0.17 | -0.03 | -0.10 | -0.03 |
| 10. As in (7.), but with $\beta_{2}=0$ | $\beta_{1}$ | -0.24 | 0.00 | -0.10 | 0.00 |
|  | $\beta_{2}$ | 0.00 | 0.00 | 0.00 | 0.00 |
| 11. As in (7.), but with $\beta_{1}=\beta_{2}=0$ | $\beta_{1}$ | -0.01 | -0.01 | -0.01 | -0.01 |
|  | $\beta_{2}$ | 0.01 | 0.01 | 0.01 | 0.01 |
| 12. As in (7.), but with $\lambda=0$ (MCAR) | $\beta_{1}$ | -0.11 | 0.00 | 0.00 | 0.00 |
|  | $\beta_{2}$ | -0.17 | -0.08 | -0.08 | -0.08 |

Abbreviations: Corr = correlation, MCAR = missing completely at random, MVNI = multivariate normal imputation, FCS = fully conditional specification.
